# Molecular insights into aggregate speciation of diglycolamides for efficient extraction of rare earth elements

**DOI:** 10.1039/d5ra03801d

**Published:** 2025-09-11

**Authors:** Kaustubh P. Bawankule, John A. Howarter

**Affiliations:** a School of Materials Engineering, Purdue University 701 West Stadium Avenue West Lafayette IN 47907-2045 USA; b School of Sustainability Engineering and Environmental Engineering, Purdue University 500 Central Drive West Lafayette IN 47907 USA howarter@purdue.edu

## Abstract

*Hypothesis*: Diglycolamides (DGAs) are widely employed as solvation extractants in industrial lanthanide separations. In solvent extraction, reverse aggregation enables these extractants to form complexation sites that significantly influence extraction yield. Although extraction is parametrically controlled, its efficiency is governed by poorly characterized phenomenological behavior. Therefore, improved control over nanoscale aggregate speciation is expected to enhance extraction efficacy at the macroscale. A multiscale framework can thus be applied to rationalize aggregation phenomena under process-relevant conditions, facilitating development of strategies to modulate solvent-phase structuring and thereby optimize extraction efficacy. *Experiments/simulations*: Aggregation tendency of DGAs is enhanced by polar infiltration from aqueous phase. To mechanistically analyze this behavior, a systematic multistage investigation was conducted on the reverse aggregation of *N*,*N*′-dimethyl-*N*,*N*′-di(*n*-octyl)diglycolamide (DMDODGA), a low-lipophilicity DGA, in *n*-dodecane over a representative experimental range of nitric acid and water concentrations, using molecular dynamics (MD) simulations. *Findings*: Cluster dynamics exhibit exponential decay, consistent with Smoluchowski's formalism. Aggregates are metastable, with nitric acid acting as a chaotropic agent at low concentrations and shifting to a kosmotropic role at higher levels. Counterions with greater solvating character in the Hofmeister series tend to favor the formation of small to intermediate aggregates. Hydrogen bonding enables water to consistently function as a kosmotropic agent, promoting the formation of larger assemblies. Free energy analysis indicates that systems with narrower cluster distributions and higher nitric acid-to-water ratios exhibit greater aggregate stability.

## Abstract 2

Surfactant based extraction optimization is governed by physicochemical phenomena occurring across multiple length scales. Effective control of aggregate speciation at the nanoscale has implications for enhancing macroscale separation efficiency.

## Graphical abstract

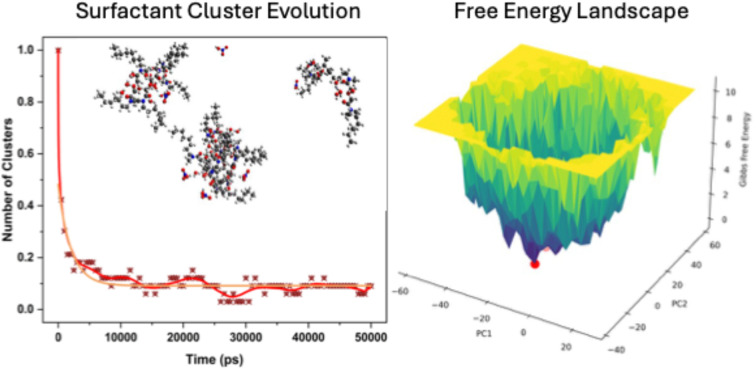

## 1 Introduction

Environmental sustainability within the chemical sector necessitates the development and optimization of separation and recycling methodologies.^1,2^ Design innovation and process intensification in standardized separation techniques are also crucial for the hydrometallurgical industry, particularly in the selective recovery of rare earth elements (REEs), which remains a fundamental challenge in separation science.^1–3^ In this context, advancements in predictive modeling and technological innovations are essential for optimizing recycling and extraction processes.^4^

Solvent extraction is a well-accepted hydrometallurgical technique for REE separation from aqueous media, known for its industrial scalability and inter-process compatibility.^1,3,5,6^ It operates on the principle that the extractant, dissolved within the organic diluent, enables the transfer of lanthanide cations from the aqueous to the organic phase. Among the most widely used extractants are organophosphorus acids, which, despite their broad application, exhibit low selectivity for adjacent lanthanides (Ln) and are highly sensitive to variations in pH and temperature.^7–9^ They rely on a pH-swing mechanism that consumes acid and base throughout the process, leading to large volumes of secondary waste.^3,10^ These limitations can be addressed by employing solvation or neutral extractants, wherein fluctuations in coanion concentration govern the equilibria between extraction and stripping phases.^3,10^ The diglycolamides (DGAs) are neutral, lipophilic, tridentate oxygen-based complexants^11^ that offer a promising alternative to acidic extractants.^10^ Dually functionalized DGA derivatives exhibit relatively high extraction strength and selectivity for lanthanides.^10^

However, DGAs are in general susceptible to phase disengagement during LLE, regardless of their nature or the diluent used.^12^ Aggregates of different magnitudes emerge as precursors to this behavior.^13,14^ As extraction stage is strongly dependent on aggregation, a deep understanding of solvent phase structuring becomes crucial for controlling its properties to facilitate accurate modeling and, ultimately, optimize the process.^4,15^ Nonetheless, organic phases are complex, organized fluids; optimizing their extraction properties requires a supramolecular approach that accounts for higher-order effects beyond traditional coordination chemistry.^16^ Standard macroscale methods like graphical slope analysis (GSA) and Job's plot, commonly used for stoichiometric determination, are limited in modeling solvent extraction, as the organic diluent phase cannot be fully described by simple equilibrium reactions due to the distribution of aggregates.^16^

In lanthanide recovery *via* DGAs, multicomponent aggregates (without Ln ions) act as ‘metal-free receptacles,’ primarily comprising DGA extractants, protic acid, and water molecules.^13,14,17,18^ The aqueous phase, often treated as a large reservoir, facilitates the migration of protic acid and water across the liquid–liquid interphase, shifting the aggregation equilibrium to favor aggregate formation in line with Le Chatelier's principle.^17^ Given the rapid timescale of aqueous partitioning into the organic phase, these heterogeneous clusters are often studied as baseline structures for lanthanide transfer in the literature.^11,13,14,17–19^

Aggregates are non-uniform in size and display distribution, necessitating a multi-equilibria approach to accurately capture their speciation in complex fluids.^20^ Most extraction formulations operate in a dilute aggregation regime and thus overlook non-electrostatic interaggregate interactions, though these effects are still relevant.^1,4^ Additionally, process models often exclude nonselective higher-order colloidal effects, which remain non-negligible under conditions typical of industrial-scale systems.^4^ Recent findings suggest that aggregate dynamics within the organic phase is largely responsible for these interactions, which underscore the need for comprehensive characterization of diglycolamide clustering, with the ultimate goal of developing strategies to control aggregate speciation and thereby enhance metal recovery.^16^

Small-Angle Neutron Scattering (SANS) and Small-Angle X-ray Scattering (SAXS) can be useful for characterizing extractant clusters. However, their effectiveness is limited by the need for model-fitting to interpret scattering profiles and simplifying assumptions about aggregate morphology.^13,14^ The sensitivity of SAXS to electron densities predominantly highlights the subnanometer cores of aggregates, often overlapping with solvent signals in the high-Q region, which complicates solvent subtraction and fitting.^16^ In contrast, SANS provides a more complete scattering profile but introduces uncertainties in core size determination.^16^ Moreover, the inherent lability and dynamic nature of aggregates add further challenges to their experimental characterization.^13,14,18^ Navigating these challenges necessitates a multiscale modeling framework as the only viable approach.^1^ Density Functional Theory (DFT) serves to identify potential interaction sites and assess the stability of extractants within complex fluids. Classical molecular dynamics (MD) simulations, with scattering techniques providing supplementary insights, offer a robust framework to probe solvent-phase structuring in multicomponent biphasic LLE systems.^20^ Insights gained about speciation can then be used to elucidate aggregation energetics by adopting a phenomenological framework at the mesoscopic scale.^1,21^

The principal objective of this investigation is to bridge chemical engineering with a molecular-level understanding of aggregation phenomena using fundamental scientific models that are broadly applicable to process intensification in separation science. Within this scope, the investigation aims to provide mechanistic insights into aggregate speciation, with an emphasis on optimizing extraction efficiency through improved molecular-level understanding. We explore whether insights from cluster characterization, even within a minimal compositional space, can effectively scale across length scales, ultimately guiding strategies to control aggregate speciation for optimizing industrial-scale LLE. To this end, DMDODGA—a diglycolamide (DGA) with one of the lowest lipophilicities^22^—is employed as a representative extractant of the DGA class to elucidate aggregation phenomena and address a broader scientific audience. A comprehensive, probability-based cluster characterization is performed in an *n*-dodecane matrix with aqueous nitric acid, spanning a range that reflects experimentally observed polar residue levels in the organic phase. The study also seeks to elucidate the driving force underlying aggregation dynamics that govern speciation behavior in industrial-scale LLE systems. Complemented by cluster distribution analysis, this approach examines the distinct yet poorly understood role of polar species in speciation profiles and suggests strategies for modulating solvent-phase structuring. An ancillary objective is to develop a statistical mechanics model that integrates probabilistic cluster occurrences with supramolecular colloidal assembly principles to predict cluster stability through free energy calculations. Identifying and correlating dominant cluster sizes with system composition could help limit large clusters, reducing the tendency for phase disengagement. While particularly applicable to hydrometallurgical separations, this model is designed for wider application across various fields. In a broader perspective, our insights hold significant implications for the design and intensification of separation processes for f-block elements, highlighting the importance of tuning speciation to obtain greater control over the extraction processes.

## 2 Methodology

The OPLS-AA forcefield refitted for the Fourier torsional coefficients along with the combining rule was used for HNO_3_, *n*-dodecane, DMDODGA.^23^ The TIP3P model was employed for water due to its compatibility with this forcefield. Density Functional Theory (DFT) optimization of DMDODGA was performed using Gaussian 16 at the B3LYP/6-311+G(2d,p) level of theory, without imposing symmetry constraints.^24^ The optimized geometric configuration was validated as true minima on the potential energy surfaces by vibrational frequency analysis at the same level of theory. NBO analysis was employed for computing the atomic partial charges of DMDODGA *via* summation over natural atomic orbitals. Separately, geometric combining rules within OPLS procedures were applied to derive the nitric acid parameters. At low nitric acid concentrations, DGAs behave as Lewis bases through their amidic moieties and preferentially interact with the undissociated form of nitric acid, forming molecular adducts.^25^ This behavior justifies the use of neutral HNO_3_ in the present simulations. Although nitric acid can dissociate under certain conditions, prior studies have shown that such speciation has a negligible effect on the resulting cluster distribution and morphology at the concentrations considered here.^18^ Therefore, to enable consistent comparison across diluent systems while minimizing unnecessary complexity, the dissociated form was not included in the simulations. All MD simulations were conducted using the GROMACS 2021.3 software suite within a framework of 3D cubic periodic boundary conditions (PBCs). The compositional space for the simulated systems, as detailed in Table 1, was adopted from the literature.^13^ These compositions correspond to experimentally observed polar concentrations that remain largely invariant under aqueous equilibrium conditions, consistent with industrially relevant extraction scenarios. While the compositional basis was adapted from a prior TODGA-based simulation study, the selected concentration ranges are consistent with reported values for organic-phase water and nitric acid in TODGA systems, which serve as a rational proxy given the limited experimental data for DMDODGA. Due to structural similarities in the diglycolamide core and comparable coordination behavior, TODGA-based data provide a reasonable reference point for constructing physically meaningful DMDODGA simulation conditions. Available data suggest that phase separation in DMDODGA systems requires substantially higher polar solute concentrations than those considered here. This context is provided to transparently distinguish our modeling assumptions and to reinforce the physical relevance of the chosen systems. Based on these design considerations, the following simulation framework was implemented. A series of systematically designed multi-level simulations were performed, with each successive stage representing enhanced complexity. Six discrete compositions were simulated overall, each characterized by different polar compositions, to analyze their impact on the spatiotemporal cluster dynamics of DMDODGA. DMDODGA concentration was uniform across all compositions, while the effects of protonic influences were assessed through modeling systems with acid concentrations varying between 0.04 M to 0.1 M, coupled with aqueous concentrations spanning from 0.07 M to 0.45 M.

**Table 1:** Composition profiles of simulated systems: the first column enumerates the system indices. The subsequent columns display the molar concentrations of DMDODGA, HNO_3_, and H_2_O, with the corresponding number of molecules listed above each concentration. The final column indicates the number of molecules of the organic diluent, *n*-dodecane

System index	DMDODGA	HNO_3_	H_2_O	*n*-Dodecane
1	10	4	8	400
	0.1	0.04	0.07	
2	10	4	16	400
	0.1	0.04	0.15	
3	10	8	16	400
	0.1	0.08	0.15	
4	10	12	24	400
	0.1	0.11	0.23	
5	10	8	32	400
	0.1	0.07	0.30	
6	10	12	48	400
	0.1	0.11	0.45	

To prepare a system with a non-aqueous diluent, a simulation box was initialized with 400 randomly inserted *n*-dodecane molecules, and the density was adjusted to 0.7495 kg L^−1^.^26^ Energy minimization was performed using both steepest descent and conjugate gradient algorithms, followed by thorough *NVT* equilibration to eliminate residual molecular ordering under PBCs, conducted over 5 ns with a time step of 1 fs at 298.15 K. A controlled insertion of 10 DMDODGA molecules was performed within a pre-equilibrated *n*-dodecane configuration, followed by steepest descent minimization with a maximum force convergence threshold of 100 kJ mol^−1^ nm^−1^. Assuming shorter conformational autocorrelation times, DMDODGA molecules were restrained, followed by equilibration of the system under the *NPT* ensemble for 20 ns until density convergence was achieved. This equilibrated configuration was used as the basis for constructing all subsequent systems, wherein varying numbers of water and nitric acid molecules were added according to the compositions detailed in Table 1. Both steepest descent and conjugate gradient algorithms were employed to minimize the energy gradient of the system configurations below 100 kJ mol^−1^ nm^−1^. All systems underwent thorough equilibration over 15 ns in two phases. The first phase involved a 5 ns equilibration using the velocity-rescaling thermostat with a time constant of 0.1 ps and the Berendsen barostat with a pressure coupling constant of 1 ps. The second phase consisted of an additional 10 ns equilibration prior to the MD simulation, utilizing the Nosé–Hoover thermostat with a time constant of 1 ps and the Parrinello–Rahman barostat with a time constant of 5 ps. Production runs were conducted for 50 ns following the release of position constraints, with the Nosé–Hoover thermostat and Parrinello–Rahman barostat maintaining the same time and pressure coupling constants as during prior equilibration. Each system configuration was simulated with an integration time step of 2 fs. The cut-off distance defining the real space was set at 1.2 nm, while reciprocal space calculations employed cubic interpolation with a 0.16 nm grid spacing. Long-range electrostatics exceeding the cut-off threshold were computed using the particle mesh Ewald (PME) summation in all three dimensions. Non-bonded potentials were modified *via* shift functions from 0.9 nm to the cut-off, ensuring energy conservation by maintaining continuous derivatives at the cut-off radius. Errors resulting from these modifications are typically smaller than integration errors and considered negligible. Bonds involving hydrogen were confined using LINCS with a fourth-order expansion. All simulations were conducted within the isobaric isothermal *NPT* ensemble, implemented with a leapfrog Verlet integrator at 1 bar and 298.15 K. Although residual size effects may exist, we have assumed them negligible within the context of qualitative trend analysis. Additional methodological details, including theoretical cluster characterization and experimental procedures, are provided in the SI.

## 3 Results and discussion

### 3.1 Cluster distribution analysis

**Fig. 1:**
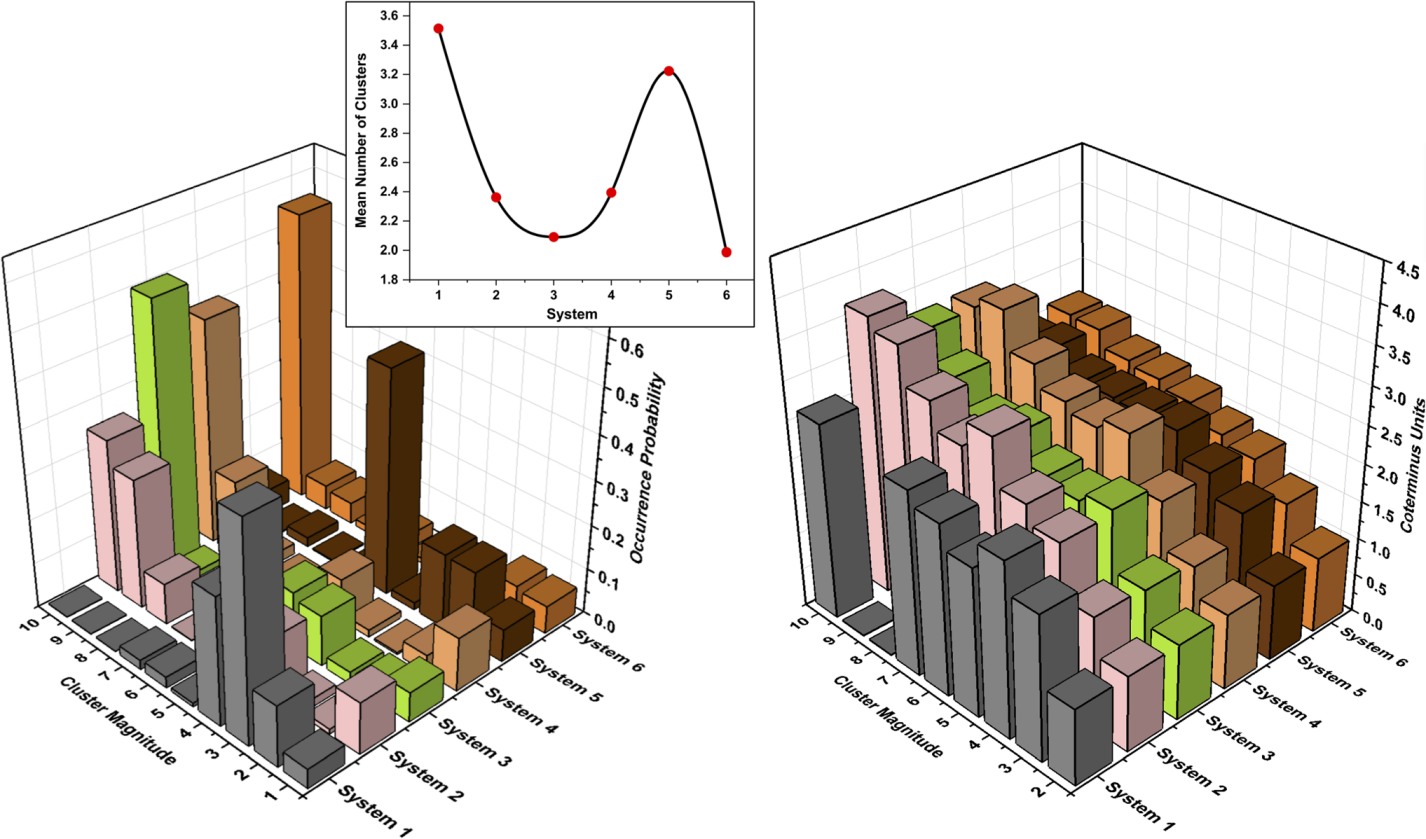
(Left) Probability of occurrence for various cluster magnitudes and the mean number of clusters (inset) across systems, highlighting distinct aggregation behaviors. (Right) Mean coterminus units for cluster magnitudes across systems.

The cluster distribution, illustrating the occurrence probability as a function of aggregate size, along with the mean number of clusters (inset), is depicted on the left in Fig. 1. The mean number of aggregates exhibits substantial variation across systems, primarily influenced by polar composition. In system 1, DMDODGA predominantly exhibits configurations ranging from monomeric to tetrameric under conditions of reduced polarity. The probability remains substantial up to a cluster size of four, with 48.4% as trimers and 28.5% as tetramers, while higher-order clusters are infrequent. In system 2, DMDODGA primarily exists as monomers and supramolecular clusters, ranging from tetra- to decameric species. Nona- and decameric aggregates are most prevalent, with a cumulative probability of 63.8%, while tetra- and octameric clusters contribute 21.8%, with the remainder mostly as monomers. The primary structural forms identified in system 3 include monomeric, tetra-, penta-, and decameric configurations. Decameric aggregates prevail with a 60% occurrence, while tetra- and pentameric forms account for 24%, and monomers occur infrequently at 6.6%. In system 4, DMDODGA typically occurs as monomers, along with penta-, nona-, and decameric supramolecular assemblies. The occurrence probability peaks at 68.6% for nona- and decameric assemblies, while pentamers and monomers contribute 9.3% and 12%, respectively. The structural distribution in system 5 is dominated by monomers, dimers, and, most notably, pentamers. Pentameric clusters dominate with a probability of 50.5%, while di- and trimeric configurations account for 32.8%. System 6 is mainly characterized by nona- and decameric supramolecular assemblies, along with mono- and dimeric forms. The probability of occurrence is notably high for decameric clusters, at 65%, while monomers and dimers collectively contribute 13.1%. Ancillary aggregates are minimal and remain transient throughout the systems. To contextualize these observations, a reference simulation containing 10 DMDODGA and 400 *n*-dodecane molecules was also analyzed. In this polar-free system, the mean number of aggregates was ∼6.0, with monomeric and dimeric forms accounting for ∼70% of the population, while transient trimers—intermittently observed throughout the 50 ns trajectory—comprised the remainder. No higher-order aggregates were formed, indicating that pure DMDODGA exhibits only weak self-association under apolar conditions. This baseline highlights the essential role of co-extracted water and nitric acid in promoting and stabilizing larger supramolecular clusters.

In general, a positive correlation was observed between aggregation propensity and dipole moment, suggesting that non-covalent interactions strengthen with increasing molecular polarity and polar content. Specifically, the amphiphilic nature of DMDODGA—with hydrophilic carbonyl and ether oxygens—was observed to promote interactions with polar residues, thereby facilitating aggregate formation.^19^ Aqueous dilution enhanced this behavior, as water molecules bridged DMDODGA residues through hydrogen bonding. Water acts as a network facilitator, contributing to cluster stabilization and promoting local structural ordering, consistent with its kosmotropic character. In contrast, a twofold reduction in acid concentration from system 3 to 2 decreased the extent of clustering and broadened the distribution of higher-order aggregates, while the equilibrium monomer concentration approximately doubled, stabilizing around 12%. This behavior reflects a nuanced chaotropic effect of nitric acid in modulating the formation of low- to intermediate-sized clusters.

More broadly, the balance between water's self-solvation and its disruption by nitric acid appears to govern the size and distribution of aggregates. At lower concentrations, water bridges extractant molecules; however, with increasing concentration, it favors self-solvation, thereby promoting the formation of larger aggregates. Nitric acid disrupts this self-solvation by reorganizing the hydrogen-bond network,^14^ redirecting water toward its multiple hydrogen bond acceptor sites, which in turn promotes the broadening of the aggregate distribution (system 2 *vs.* 1). These competing effects collectively influence the internal structure of the aggregates. The polar core appears loosely confined, likely due to multidentate interactions between nitric acid and the extractant.^10,27^ This behavior can be attributed to nitric acid's preferential association with both the polar groups and alkyl chains of DMDODGA, which leads to its partial exclusion from the core region.

Taken together, these results illustrate how the subtle interplay between water and nitric acid concentrations modulates cluster size distribution and polar core structuring—providing mechanistic insights for understanding speciation behavior in DGA-based extraction systems.

### 3.2 Cluster dynamics

Cluster dynamics, particularly in the context of DGAs usage in LLE, still remain unclear, a gap that limits the development of predictive models and process optimization strategies.^16,19,28^ While several studies have examined aspects of supramolecular aggregation, a specific phenomenological framework for DGAs remains underdeveloped, complicating efforts to systematically modulate cluster distributions and mitigate third-phase formation. To address this gap, we employed the Smoluchowski formalism,^29,30^ adopting a colloidal aggregation perspective to establish a theoretical basis for understanding the mechanism governing aggregate formation. The derivation presented herein is based on the framework outlined in,^31^ with further details available in the SI. Notably, while DMDODGA – a representative DGA with high aggregation tendency – serves as a primary model for this analysis, the framework derived herein is expected to extend to a wider range of DGAs, underscoring its general applicability within this class of extractants.

**Fig. 2:**
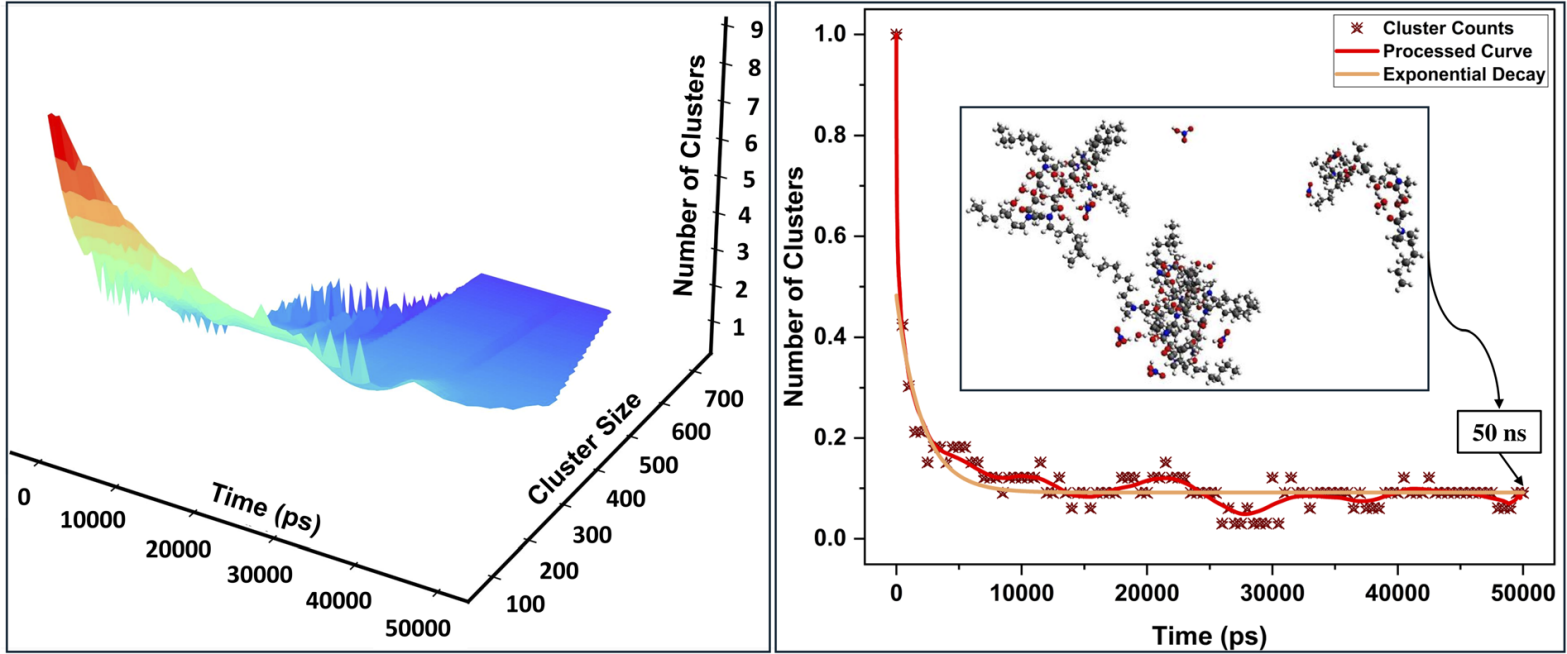
( Left) Temporal evolution of cluster size and count in system 5, exhibiting a systematic increase in size, with a corresponding decline in number. (Right) Normalized cluster count over time; red curve represents the processed trend, beige curve depicts an exponential decay model (3.5 Å proximity criterion for cluster identification);^13^ inset shows MD snapshot at 50 ns.

Within this construct, the temporal evolution of the cluster size *k*, with concentration *c*_*k*_(*t*), can be represented by the following master equation:1
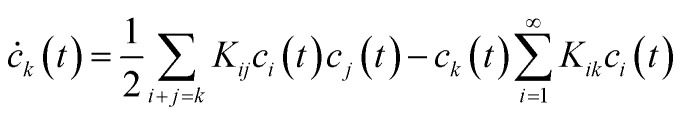
where *ċ*_*k*_(*t*) denotes the time derivative of *c*_*k*_(*t*). *K*_*ij*_ and *K*_*ik*_ are the reaction kernels, representing the frequency likelihood of cluster magnitude *k* formation. *c*_*i*_(*t*), *c*_*j*_(*t*) indicate the concentration of *i*-mers and *j*-mers, respectively. Based on the observed aggregation tendency of small-sized clusters (Fig. 2, inset), a sum kernel was used to model the dynamics assuming their colloidal aggregate-like behavior. The sum kernel, indicative of clustering propensity in general, accounts for the aggregation of *i*-mers with *j*-mers in the formation of (*i* + *j*)-mers clusters.2
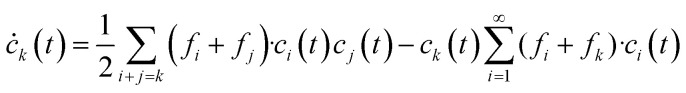
where *f*_*i*_ and *f*_*j*_ represent size-dependent aggregation rates, each proportional to the respective cluster magnitudes *i* and *j*.

The full derivation, including the transformation to normalized cluster concentrations and integration using an almost-exponential ansatz, is provided in the SI. The final closed-form expression for the instantaneous concentration of clusters of size *k* is:3
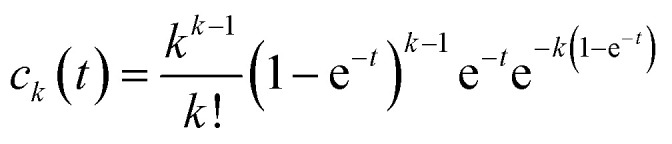


Summation over all cluster magnitudes yields the total number of instantaneous clusters, *N*(*t*), corresponding to the first moment, which is equivalent to e^−*t*^:4
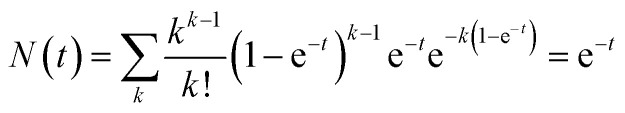


Analysis of simulation data indicates an exponential decline in cluster count (Fig. 2, right), corroborating Smoluchowski's aggregation model, which predicts a shift toward fewer, larger aggregates through exponential decay kinetics, as illustrated in Fig. 2, left. This analytical solution provides qualitative insights into aggregate behavior within a complex organic fluid, suggesting, in the ideal asymptotic limit (*t* → ∞), phase disengagement (third-phase formation) would occur as the number of clusters approaches zero (*N*(*t*) → 0) in the solvent phase. This phase separation tendency is further supported by the earlier use of additive kernel, *K*_*ij*_ = (*f*_*i*_ + *f*_*j*_), wherein new cluster formation rates scale proportionally with the cumulative size of constituent clusters. Over time, a gradual shift toward the cluster–cluster aggregation (CCA) regime becomes evident, leading to the emergence of seemingly stable, larger clusters at extended timescales. This transition appears entropy-driven, as weak interaggregate associations allow the system to access a broader range of configurations within the simulation timescale, thereby increasing configurational entropy and favoring the formation of larger aggregates.^32^

The apparent stability of aggregates and their persistent inclination toward a third phase reinforce their metastable nature^13,14^—an insight essential for developing effective strategies to control aggregate speciation. Cluster metastability is primarily attributed to long-range interactions, which result from higher-order colloidal effects largely influenced by polarized species.^21,28^ Among such polarized species, nitric acid—due to its distributed electron cloud and high polarizability—acts as a chaotropic (disorder-inducing) salting-in agent at low concentrations, effectively mitigating long-range cluster–cluster interactions in the solvent phase.^14^ At higher concentrations, however, its role appears to reverse, strengthening collective interaggregate interactions and behaving more like a kosmotropic (order-inducing) agent, as observed in systems 4 and 6.^13^ In contrast, water consistently behaves as a kosmotropic agent, with hydrogen bonding networks promoting the formation of larger aggregates. Given this behavior, maintaining low nitric acid concentrations favors the formation of small to intermediate aggregates, which are generally considered more conducive to efficient metal extraction in large-scale LLEs.^3,27^ This suggests that, under reduced acidity in the solvent phase, acids with stronger chaotropic character may allow the extraction process to approximate simple coordination complex formation, with nitrates likely to exhibit higher efficacy than other anions, such as chlorides and sulfates, when evaluated in the context of the Hofmeister series.^22,28^ On the other hand, adding a salting-out agent in the ‘aqueous phase’ can promote metal ion migration to the organic interphase; however, in such cases, extraction modeling must carefully account for salting-out agent activity and adopt a multiple equilibria approach to enhance process efficiency at macro scale.^20^

### 3.3 Structural correlations

#### 3.3.1 DMDODGA–DMDODGA interactions

Aggregates are commonly modeled as pseudophases in biphasic multicomponent extraction systems, yet their speciation is often excluded from process models due to inherent complexity.^33^ While such omission is reasonable under dilute conditions, it becomes inadequate at higher metal and extractant loadings typical of industrial systems.^15^ This exclusion stems from the multicomponent, disordered, and metastable nature of aggregates, which complicates their incorporation into complexation frameworks.^13,15^ Nevertheless, aggregates exert significant long-range effects that can be addressed *via* soft-matter colloidal approaches.^21^ Although complexation energy primarily drives extraction, these collective interactions facilitate tuning of cation recovery.^21,33^ While long-range structural correlations are recognized, their relationship to aggregate morphology and potential for enhancing speciation control remains largely unexplored.^13,14,18,34^ The objective is to qualitatively understand the structural correlations and the role of co-extracted solutes—water and nitric acid—in guiding strategies for improved speciation control.

**Fig. 3:**
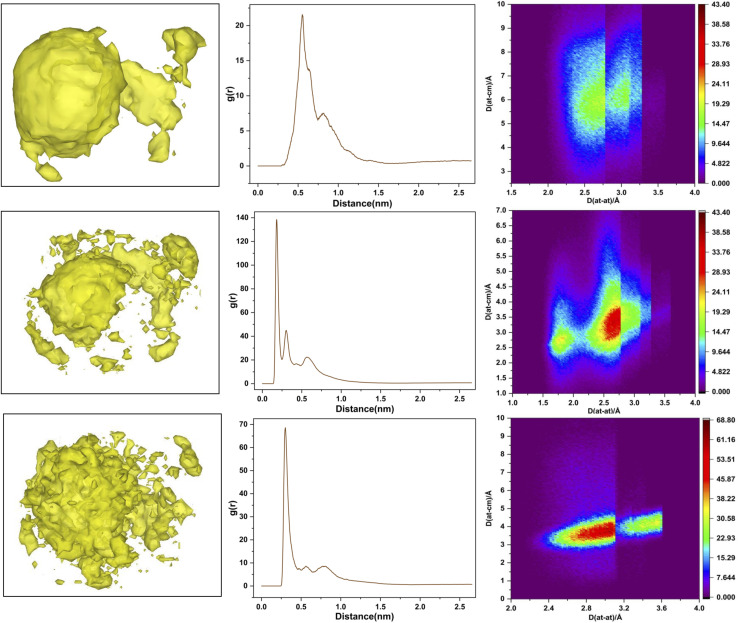
Structural correlations within system 5: the left panel shows spatial distribution functions (SDF) for DMDODGA–DMDODGA, DMDODGA–water, and DMDODGA–nitric acid interactions, illustrating spatial correlations. The center panel presents radial distribution functions (RDF), indicating variations in local pair correlations. Please note that RDFs and SDFs use the COM of three oxygens (two carbonyls and one ether) of each DMDODGA as the reference center (excluded for visual clarity.) The right panel displays first-shell analysis (FSA), providing insights into molecular interactions.

To probe DMDODGA interactions within aggregates, radial distribution functions, *g*(*r*), were analyzed to quantify time-averaged coordination metrics. These metrics were correlated with coterminus units, which were calculated using the cluster identification algorithm described in the SI, following an approach analogous to persistent homology in topological analyses.^14,34^ The mean coterminus units for different cluster sizes are shown (see Fig. 1, right), with corresponding RDF plots for each system provided in the SI (Fig. S4). To visually illustrate these correlation features, a representative example from system 5 is shown in Fig. 3, which integrates spatial and radial distribution data along with first-shell analysis.

In the system with minimal polar solute concentration (system 1), the coordination number—obtained by integrating the radial distribution function up to the first minimum—was 1.92. This value closely corresponds to the average number of coterminus units in dominant tri- and tetrameric species, indicating a recurring structural motif throughout the simulation. Such aggregate uniformity, characterized by low polydispersity and a higher proportion of trimeric forms, suggests that ‘the aqueous nitric acid concentration in equilibrium with this organic phase’ would favor more efficient lanthanide extraction, consistent with prior reports on lanthanides' preference for trimeric complexation in LLE systems.^3,10,22^

In contrast, system 6 – with the highest polar content – exhibits a cumulative coordination number of 2.585, corresponding to the mean coterminus units of a decameric aggregate, also identified as the most probable configuration in cluster analysis. This indicates that structural correlations extend beyond the primary coordination shell, even in disordered assemblies. DMDODGA predominantly resides within the primary shell,^13^ though higher polarity may shift it toward a weaker secondary shell, as reflected by a subdued secondary RDF peak in system 6. Solvent-accessible surface area (SASA) of decameric aggregate remains low but fluctuates due to the dynamic interplay between polar solutes and extractant chain conformations that limit diluent access to the polar core.^4,21^ Reducing water content could suppress these fluctuations, increase SASA, and lower aggregation numbers. Additionally, introducing salting-out agents may further confine the polar core, favoring smaller, more efficient aggregates for metal uptake.

It should be noted here that stoichiometry in extraction experiments is typically estimated using the slope analysis or Job's plot—an approximation that can vary, allowing for potential exchange of extractants between the primary and secondary coordination spheres, depending on system conditions.^1,16^ This notion is further supported by observed migration of extractants between coordination spheres during simulation (*e.g.*, system 4 and 6). In light of these observations, current practices in industrial process flowsheet modeling—which typically emphasize complexation number—overlook important aspects of extraction behavior. Instead, aggregation number should be considered a more relevant descriptor for capturing the underlying dynamics, particularly in systems where exchange between coordination spheres cannot be overlooked.^15,17,33,35^ This complexity is evident even in our simplified system, which, despite its minimal setup, still demonstrates such intricate behavior.

In general, increasing water concentration is observed to strengthen short-range soft interactions within the primary coordination sphere of DMDODGA. Higher water concentration results in the swelling of the polar core in aggregates.^1,13,18^ This swelling raises the local dielectric constant.^1,4^ As a result, the first-shell pair correlation density of extractants increases, accompanied by a weak secondary RDF peak—features that reflect greater aggregation extent and higher polydispersity, as evident in system 2 relative to system 1, and together contribute to aggregate stabilization.

In contrast, higher nitric acid concentrations (*e.g.*, systems 4 and 6) shift its role toward a stabilizing agent, suppressing long-range correlations and promoting compact cluster formation through enhanced dipole–dipole and hydrogen bonding interactions.^14^ This transition is marked by the disappearance of the RDF second peak and broadening of the first peak from system 2 to 3. Thus, while it is apparent that nitric acid modulates cluster formation at lower concentrations (system 1, 2), its role shift toward stabilizing larger assemblies at higher concentrations,^13^ though further stability analysis is required to support this interpretation. This dual role is observed to be ‘highly sensitive to the concentration’ of nitric acid, where even slight variations affect its behavior—initially promoting smaller clusters at lower concentrations but stabilizing larger assemblies once a threshold concentration is reached.^13,14,18,22^

#### 3.3.2 DMDODGA–water interactions

RDF plots for DMDODGA–water interactions are in the SI (Fig. S5). The RDF profiles exhibit a consistent pattern in the spatial correlations of water around DMDODGA, independent of composition. Variations in water concentration do not affect its local distribution within the aggregates. Notably, this structural order persists even within the ‘dispersed’ hydrophilic core, indicating that the observed mobility within the core is possibly due to site-hopping phenomena, wherein water molecules transiently shift between neighboring coordination sites without disrupting overall structural order.^1,18,36^ This is supported by the dynamic intra- and inter-sphere exchange of water molecules within, and migration to, the second and third coordination spheres observed during simulation. An important conclusion here is that, contrary to the general notion of water being more dispersed with variable concentration within aggregates, the coordination metric of water remains consistently invariant across compositions. This persistent correlational order suggests that water's spatial organization within aggregates remains largely composition-invariant, which can streamline the calculation of chemical potentials in mesoscopic models of soft-matter colloidal interactions during extraction processes.^20,21^

##### 3.3.2.1 First shell analysis

FSA maps for DMDODGA–water interactions are provided in the SI (Fig. S6). Two distinct red regions emerge. The first, a narrow and intense zone at 0.18 nm, reflects strong hydrogen bonding between water and the highly electronegative amide oxygens of DMDODGA, consistent with a sharp RDF peak. The center-of-mass distribution of water is confined to 0.26–0.29 nm, attributed to a homo-bidentate orientation where both water hydrogens interact only with DMDODGA's amide oxygens. In contrast, the second red region appears broader (0.25–0.3 nm), corresponding to the secondary hydration shell. Here, the water center-of-mass extends up to 0.37 nm due to hetero-bidentate orientations—one hydrogen interacting with DMDODGA's alkyl or amide groups, and the other with nitric acid. This diffuse behavior arises from a shift in water orientation, influenced by the hydrophobic alkyl chains of extractants and hydrogen bonding with nitric acid, which also reaffirms the previously discussed role of nitric acid in modulating the speciation of aggregates.^18,36^

#### 3.3.3 DMDODGA–nitric acid interactions

RDF plots and corresponding FSA profiles for DMDODGA–nitric acid interactions are provided in the SI (Fig. S7 and S8). The RDF shows a primary peak near 0.3 nm, spanning the 0.25–0.4 nm range, which aligns with two high-probability regions within the single coordination shell in the FSA map. The first region (0.26–0.31 nm), accounting for ∼70% of interactions, reflects strong hydrogen bonding between nitric acid and DMDODGA's oxygen atoms. The second region (0.32–0.36 nm) corresponds to weaker interactions between nitrate oxygens and DMDODGA's alkyl groups. These interactions follow a bidentate orientation—where nitric acid simultaneously engages with both polar and nonpolar moieties of DMDODGA—resulting in a consistent directional pattern across the cluster distribution. Importantly, these structural correlations remain preserved across different systems regardless of composition, in contrast to the variability observed in water's more diffusive behavior.

These observations underscore several key insights: structural correlations among polar species persist even within seemingly disordered, labile aggregates, with spatial localization remaining consistent across compositions. However, their orientations depend critically on the hydrophilic centers of surrounding species and the conformations of alkyl chains. Additionally, long-range electrostatic interactions play an important role in aggregate stability.^21^ Neglecting the second coordination sphere could lead to substantial discrepancies between theoretical predictions and experimental results, as structural influences extend beyond the primary sphere, even in the metal-free host receptacles. Since structural correlations are invariant across compositions, higher-order intra-aggregate effects can be accounted for by incorporating discrete electrostatic coupling terms at the colloidal scale when developing mesoscopic LLE models, which can then be leveraged to control aggregate speciation.^20,21,33^ However, this integration requires slight modifications to the existing framework, particularly in calculating the chemical potential of aggregates during multiscale modeling of solvent extraction processes.^20,33^

### 3.4 Cluster stability

To evaluate the Gibbs free energy associated with aggregate formation, we applied the probabilistic invariant cluster free energy (PICFE) approach, which accounts for the influence of cluster size distribution within the system. This method focuses on system-dominant aggregates that largely control the thermodynamic stability of the overall cluster distribution. A detailed derivation and theoretical background are provided in SI. The Gibbs free energy change associated with the formation of i-sized aggregate from the *i* individual monomers is given by,5
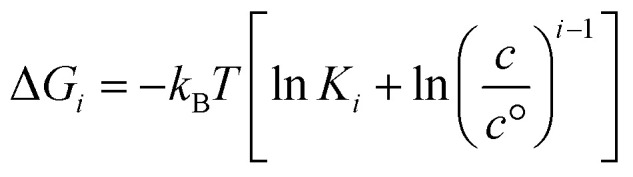
where Δ*G*_*i*_ denotes the Gibbs free energy change for an *i*-sized cluster, *K*_*i*_ is the local equilibrium association constant, and *c* & *c*° represent the system and standard reference concentration, respectively.

The applicability of this equation is limited to aggregates of uniform size, as it depends on the local association constant and concentration. However, in systems where a narrow range of larger aggregates governs the distribution—such as nona- and decamers in system 4—a broader thermodynamic perspective is needed. To address this, the framework was extended using a law-of mass action based treatment, facilitating evaluation of global free energy across the aggregate distributions to reflect overall system behavior. This extension relies on two simplifying assumptions: the first involves deriving the system association constant under the condition that the Gibbs free energy change, upon adding an additional residue, remains invariant of cluster size *i*, consistent with the commonly-used isodesmic model approximation.^13^ The second considers DMDODGA cluster probability occurrences, following a 20 ns production run assuming equilibrium, as the basis for calculating individual cluster concentrations. The derivation supporting the first assumption is briefly outlined below:*M*_*n*−1_ + *M*_1_ ↔ *M*_*n*_
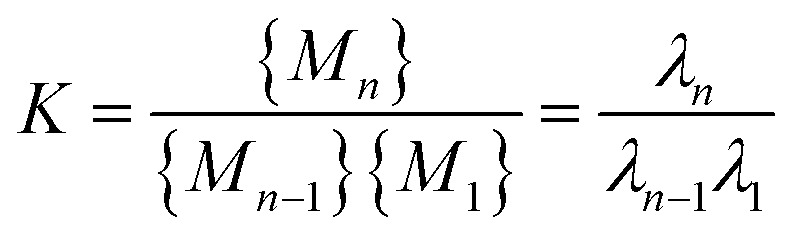
*λ*_*n*_ = *Kλ*_*n*−1_*λ*_1_*λ*_*n*_ = *K*^*n*−1^*λ*_1_^*n*^6
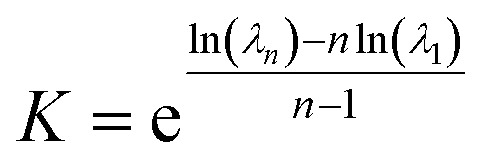
where *K* denotes the representative system association constant and *λ* represents the activity coefficient. With the parameters (*K*, *c*) now representing system-wide metrics, the PICFE approach defines the cluster size *i* as representative of the overall system through the introduction of the weighted average aggregate size. By incorporating the system association constant, weighted average cluster size, and concentrations based on the second approximation into eqn (5), the resulting formulation becomes:7
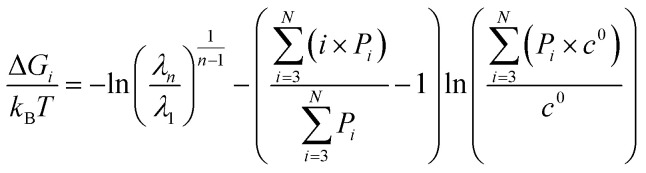
where *P*_*i*_ denotes the probability of clusters with size *i*. Probability occurrences for the respective cluster sizes are employed relative to the reference concentration *c*°.

The evaluation of the Gibbs free energy across all systems exhibits distinct stability profiles, governed by the interplay between cluster distribution and polar content. The details of the calculation are provided in the SI (Fig. S11 and S12). In general, systems with a predominant occurrence of narrowly distributed cluster sizes, such as system 3 and system 6, display relatively higher stability, as evidenced by more pronounced negative shifts in Δ*G* values. Systems with higher nitric acid-to-water ratios exhibit increased aggregate stability, except for system 4, which displays a moderately broad cluster distribution. Diluent effects also exert a notable influence on aggregate stability. In particular, system 5—characterized by one of the highest solvent-accessible surface areas (SASA)—exhibits solvent-mediated separation between clusters, which hinders long-range interaggregate interactions and leads to reduced aggregate stability. Greater solvent penetration in system 5 increases polar core curvature, which promotes solvent-mediated cluster separation and results in smaller aggregate sizes. This further underscores the crucial role of the diluent in controlling aggregate speciation. Although the present study focused exclusively on dodecane to isolate intrinsic aggregation behavior, the influence of diluent polarity on supramolecular organization is nontrivial and warrants consideration in downstream modeling.

**Fig. 4:**
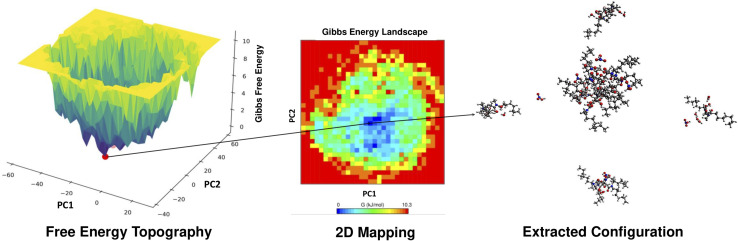
Free energy analysis of system 5: left, three-dimensional free energy landscape (3D FEL); middle, corresponding two-dimensional (2D) mapping of Gibbs free energy surface, both derived from principal component analysis, depicting the energy topography; right, extracted configuration showing a stable pentameric assembly, corroborated by its highest probability occurrence in cluster distribution analysis.

The PICFE model results were evaluated against free energy landscapes (FELs) to assess consistency and elucidate the influence of cluster distributions and structural correlations on stability profiles. The PICFE model and FEL analysis offer complementary routes to characterize the free energy profile of DMDODGA aggregates. PICFE captures system-level thermodynamic favorability based on dominant cluster populations, while FELs provide a coarse-grained configurational view of the energy landscape derived from MD trajectories. Energy basins observed in FELs align with aggregate stability trends predicted by PICFE. The 3D FELs and 2D mappings for all systems are provided in the SI (Fig. S9 and S10), with system 5 shown as an example in Fig. 4. Systems 1, 3, and 6 exhibit concentrated low-energy basins with well-defined boundaries in the FELs, consistent with the PICFE model's prediction of higher Gibbs free energy changes for these systems. While system 1 also shows a concentrated low-energy basin, its boundaries are comparatively less defined than those in systems 3 and 6, which may be attributed to the coexistence of predominant trimeric and tetrameric clusters, unlike the uniform cluster sizes observed in systems 3 and 6. This subtle deviation is also reflected in the PICFE results, indicating slightly lower stability for system 1. In contrast, systems 2, 4, and 5 display more dispersed free energy landscapes, reflecting multiple accessible energy states, consistent with the PICFE model's prediction of lower Gibbs free energy changes. Systems 2 and 4 exhibit relatively broad cluster distributions, with DMDODGA showing a weak tendency to form secondary coordination shells, which enable clusters to explore a broader range of energetically accessible configurations on the FELs. System 5, dominated by low- to intermediate-magnitude clusters and containing the highest proportion of monomers and dimers (24%) among all systems, exhibits the most diffuse energy landscape, corresponding to the lowest Gibbs free energy change observed in the PICFE results.

The PICFE approach is compared with the Isodesmic model to assess differences in the calculation of aggregation free energy (see Fig. S15). The Isodesmic model assumes that the free energy change associated with each successive monomer addition to an aggregate is constant, regardless of cluster size.^13^ In practice, the Isodesmic model includes monomeric and dimeric species in the aggregation free energy calculation,^13^ which can lead to an overestimation of aggregate stability. Alternatively, the PICFE approach adopts a selective exclusion strategy, disregarding mono-, dimeric forms to better capture the bulk aggregation dynamics within the systems. This exclusion is justified as the extensive surface exposure of mono- and dimers to apolar *n*-dodecane diminishes their ability to sustain stable polar interactions. The overall order of magnitude of the calculated aggregation free energy for both approaches was found to be approximately 1–2 *k*_B_*T*, which is typical of weak interaction mechanisms in complex colloidal systems.^21^ Plot trends illustrate that the Δ*G* values from the PICFE model are consistently higher, or less negative, than those from the Isodesmic model across all systems. This highlights the reduced stability of aggregates calculated *via* the PICFE model, attributed to the exclusion of monomers and dimers from the Gibbs free energy calculations, thus providing a more nuanced representation of aggregation dynamics. This distinction is particularly beneficial for modeling LLE processes using mesoscopic approaches, as the free energy of transfer in typical cascade separation systems is on the order of 4 *k*_B_*T*.^1,37,38^ The overall trend suggests that larger clusters exert a dominant influence on the system's free energy landscape. It should be noted here that, while excluding mono- and dimers reduces over-stabilization, this approach adds complexity to the model and may underrepresent their transient role in early-stage aggregation, highlighting a potential limitation of the PICFE framework.

## 4 Conclusions

Diglycolamides are widely employed as solvation extractants in lanthanide separations. Their complexes with cations are typically represented using coordination models in industrial flowsheet modeling. However, recent studies have shown that these extractants form cation-free aggregates, which act as baseline structures during the extraction process.^13,14,18^ While extraction is parametrically regulated, its optimization is governed by physicochemical phenomena occurring across multiple length scales. Accordingly, effective control of aggregate speciation at the nanoscale has implications for enhancing separation efficiency at the macroscale. In this context, a multiscale modeling framework offers a means to rationalize aggregation behavior at process-relevant conditions and enables the development of strategies to control solvent-phase structuring, which can be integrated into phenomenological modeling frameworks to enhance extraction efficacy.^1,20^

To examine the factors governing speciation control at the nanoscale, molecular dynamics simulations were conducted for six DMDODGA-based systems with systematically varied H_2_O and HNO_3_ compositions, followed by analysis of cluster distribution, dynamic behavior, structural correlations, and thermodynamic stability. Cluster distribution analysis indicates a positive correlation between DGA aggregation and the total polar content in the diluent phase. Water initially acts as a kosmotropic bridging agent, shifting to self-solvation as its concentration increases. At low concentrations, nitric acid exhibits chaotropic behavior, promoting small to intermediate aggregate formation, while at higher levels, it assumes a kosmotropic role, stabilizing larger supramolecular assemblies. Although previous studies highlight nitric acid's stabilizing effect,^13,28^ our study provides the first indication of its dual behavior.

Cluster dynamics reinforces the metastable nature of DGA aggregates. The aggregates follow Smoluchowski's aggregation model, wherein the phase disengagement tendency of diglycolamides drives aggregation by enhancing configurational entropy, explaining the labile and apparently disordered nature of the clusters. Qualitative agreement with SANS/SAXS experiments supports the irregular, labile behavior of the aggregates. This interpretation is consistent with earlier findings reported,^32^ which describe similar entropy-driven aggregation behaviors in related systems. Such insights become crucial for decoupling weak interaggregate interactions during multiscale modeling of LLEs.^21^

At lower concentrations, nitric acid acts as a salting-in agent in organic phase, mitigating long-range interaggregate interactions and narrowing aggregate speciation to small and intermediate sizes, which serve as better complexation sites than larger clusters at higher acidity, thereby enhancing extraction efficacy. This pseudophase view of aggregates is consistent with Langmuir adsorption models, where aggregates are approximated as complexation sites with binding energies analogous to those in complexation.^1,39–41^ Under reduced acidity, counterions with stronger chaotropic character enable the extraction process to approximate simple coordination complex formation, with nitrates likely to outperform chlorides and sulfates in line with the Hofmeister series. Earlier studies on related DGAs show a similar trend, qualitatively supporting the interpretation of chaotropic ion influence on extraction.^28,42^ Additionally, introducing a salting-out agent in the aqueous phase may facilitate ion migration across the LLE interphase, though accurate modeling should incorporate multiple equilibria, as recently explored,^20,28,33^ to predict efficacy at the macroscale.

Structural correlations are evident within the polytopic assemblies, despite the apparent irregularity and mobility. Results indicate that structural correlations are invariant across polar compositions. This invariance allows for a more realistic representation of hydrophilic nanodomain effects in the multiscale modeling of LLEs. Current models often subsume these effects within the polar core of aggregates, typically approximated as confined aqueous electrolyte droplets^20,33^— an approach that may not fully capture intraaggregate interactions, such as nitric acid delocalization within the core.^43^

The probabilistic invariant cluster free energy (PICFE) approach was employed to evaluate cluster stability. A correlation is observed between stability and the distribution of cluster sizes, with systems featuring more narrowly distributed magnitudes demonstrating greater stability. Similarly, systems with higher nitric acid-to-water ratios tend to form more stable aggregates. The selective exclusion of monomers and dimers in the PICFE approach effectively mitigates the over-stabilization commonly associated with Isodesmic models,^13^ offering a more nuanced depiction of aggregate stability. The Gibbs free energy of aggregation, measured at approximately 1–2*k*_B_*T*, reflects the weak interaction mechanisms typical of complex colloidal systems^21^ and is consistent with previous findings on DGA variants,^13^ showing energies comparable to thermal fluctuations.

In principle, a multiscale framework can thus be applied to study aggregation across length scales, offering insights essential for speciation control. This approach holds particular value in process flowsheet modeling, where solvent-phase structuring is often represented by well-defined clusters, frequently overlooking aggregate speciation.^1,35^ The insights presented herein provide pathways for modulating aggregate distribution and can inform process models, ultimately guiding the efficient design and optimization of extraction processes.

Further investigations are needed to elucidate the effects of diluents, extractant synergism, and mineral acids on DGA aggregation, with a focus on modulating aggregate distribution to optimize solvent extraction performance. Additionally, comprehensive evaluation of REE cation influence on clustering, and the potential application of these findings to liquid–liquid extraction (LLE) systems, is required through both experimental and simulation approaches. Such studies will not only deepen our understanding of aggregation behavior but also pave the way for designing more efficient and optimized solvent extraction systems.

## Conflicts of interest

There are not conflicts of interest to declare.

## Supplementary Material

RA-015-D5RA03801D-s001

## Data Availability

The data supporting this article have been included as part of the SI. See DOI: https://doi.org/10.1039/d5ra03801d.
