# Supplementary material for: Molecular insights into aggregate speciation of diglycolamides for efficient extraction of rare earth elements
Source: RSC Adv. 2025 Sep 11;15(40):33006–17. doi: 10.1039/d5ra03801d (PMC12424397; doi:10.1039/d5ra03801d)
Supplement: RA-015-D5RA03801D-s001 [file RA-015-D5RA03801D-s001.pdf]

Supplementary Information (SI) for RSC Advances.
This journal is © The Royal Society of Chemistry 2025

Supporting Information for: " Molecular Insights into Aggregate Speciation of Diglycolamides for Efficient Extraction of Rare Earth Elements"

Kaustubh P. Bawankule,[1,*] John A. Howarter[1,2,†]

[1]School of Materials Engineering, Purdue University, West Lafayette, IN 47907-2045

[2]Environmental and Ecological Engineering, Purdue University, West Lafayette, IN 47907

†Corresponding author: John A. Howarter. Email: howarter@purdue.edu.
*Primary author.

Cluster Characterization and Analysis

Aggregate Distribution

An extensive analysis of cluster distribution and volumetric characteristics was conducted using the AGGREGATES program, which employs the statistical framework outlined in [1]. Cluster identification was based on intermolecular connectivity, with neighbor lists determined via a van der Waals (VDW) radii threshold [1]. Interaction centers were defined through shells constructed around residues using these radii, and subsequently employed to investigate cluster phenomena.

Statistical analysis of configurations sampled across the system's trajectory was conducted to estimate the mean number of clusters in each configuration:

$$x_{agg} = \frac{\sum_{i=1}^{N} k(i)}{N} \tag{1}$$

where x_{agg} denotes the mean number of clusters per configuration, with $k(i)$ indicating the number of clusters in configuration i.

Analogously, the probability of finding a residue in a cluster of size x was determined by:

$$P(x) = \frac{x.\sum_{i=1}^{N} k_x(i)}{N \cdot T} \tag{2}$$

where $P(x)$ represents the probability occurrence of clusters of magnitude x, $k_x(i)$ denotes the number of clusters with size x in configuration i, and T refers to the total number of residues in the system.

Molecular Coordination and Spatial Distribution

The characterization of molecular neighborhoods within the aggregates was performed by estimating:

$$N_\alpha(x) = \frac{\sum_{i=1}^{N} V_{\alpha,x}(i)}{\sum_{i=1}^{N} T_{\alpha,x}(i)}, \quad D_\alpha^m(x) = 100 \cdot \frac{\sum_{i=1}^{N} N_\alpha^m(i,x)}{N \cdot x} \tag{3}$$

where $V_{\alpha,x}(i)$ and $T_{\alpha,x}(i)$ represent the neighboring or coterminus units and the number of residues α in a cluster of size x in configuration i, respectively, while $N_\alpha(x)$ indicates the time-averaged number of neighbors per residue in an aggregate of size x; $N_\alpha^m(i,x)$ corresponds to the number of residues α with m neighbors, and $D_\alpha^m(x)$ denotes the distribution of the number of neighbors as a

function of cluster size.

The mean coterminus units, determined through this approach, were correlated with the coordination numbers derived from the pair correlation function to elucidate the local coordination environments. The coordination number (CN) was computed by integrating the radial distribution function (RDF), $g(r)$, up to the first minimum, with

$$\text{CN} = 4\pi\rho \int_0^{r_{\text{min}}} r^2 g(r)\, dr \tag{4}$$

where ρ is the number density, r is the radial distance, and r_{min} denotes the position of the first minimum in $g(r)$.

Volumetric Characterization

The cluster volume was determined via polyhedral decomposition, utilizing the VDW volume approach. The cluster size was derived from the mean spatial extent of the aggregates, given by:

$$R_d(x) = \frac{\sum_{i=1}^{N} \frac{\sum_{x=1}^{k} \frac{d(x)}{d_{box}}}{k}}{N} \tag{5}$$

where $R_d(x)$ is the mean maximum span of a cluster of size x, $d(x)$ indicates the longest distance between two atoms in the cluster of size x, d_{box} represents the diagonal length of the simulation box, and k refers to the number of aggregates of size x.

Additionally, the mean packing fraction of the system was calculated using the VDW volume, expressed as:

$$C_{pack} = \frac{\sum_{i=1}^{N} \frac{V_{pack}}{V_{box}}}{N} \tag{6}$$

where C_{pack} indicates the mean packing coefficient of the system, V_{pack} represents the volume occupied by all the residues in the system, and V_{box} corresponds to the volume of the simulation box.

Free Energy Landscape

System dynamics are defined by temporal variations in structural configuration, mapped to a vector space with dimension corresponding to the system's degrees of freedom (DOF). MD trajectory samples the accessible ensemble from this vector space, with the extracted configurations subsequently utilized to conduct energetic assessments informed by the energy landscape. Dimensionality reduction was performed using Principal Component Analysis (PCA) to describe system dynamics by filtering observed motions across scales through a systematic decomposition process [2, 3].

The trajectory matrix, X, with dimensions $n_{\text{configuration}} \times$ DOF, was mean-centered to X', and the covariance matrix C was computed as $C = X' \cdot X'^{\mathrm{T}}$. Eigenvalue decomposition was performed on the C-matrix to derive orthogonal collective modes, or eigenvectors v_i, each associated with its respective eigenvalue λ_i, encapsulating the system dynamics through the relationship $Cv_i = \lambda_i v_i$. These modes describe the correlated movements in inter-residue distances, where the principal two, characterized by their maximal variance, are sufficient to encompass the system dynamics. Subsequently, the first two eigenvectors, referred to as principal components (PC1 and PC2), were employed as order parameters to linearly transform the trajectory matrix. The free energy of the configurational ensemble was then quantified as a function of these order parameters relative to the most probable configuration, defining energy basins. This approach, utilizing the Boltzmann statistical framework, enables the description of aggregation phenomena via the system's free energy landscape, given by: $\Delta G = -k_B T \ln \left(\frac{P}{P_{\text{max}}} \right)$, where k_B is the Boltzmann constant, T is the temperature, and P is the probability distribution of the system as a function of the order parameter.

Analytical framework for DGAs cluster dynamics

The detailed derivation provided below is based on the framework outlined in [4] . The temporal evolution of the cluster magnitude k, with concentration $c_k(t)$, can be represented as:

$$\dot{c_k}(t) = \frac{1}{2} \sum_{\substack{i,j \\ i+j=k}} K_{ij} c_i(t) c_j(t) - c_k(t) \sum_{i=1}^{\infty} K_{ik} c_i(t)$$

where $\dot{c_k}(t)$ denotes the time derivative of $c_k(t)$. K_{ij} and K_{ik} are the reaction kernels, representing the frequency likelihood of cluster magnitude k formation. $c_i(t)$, $c_j(t)$ indicate the concentration of i-mers and j-mers, respectively.

Employing the sum kernel, $K_{ij} = f_i + f_j$, to represent the aggregation propensity in the master equation:

$$\dot{c_k} = \frac{1}{2} \sum_{i+j=k} (f_i + f_j) c_i c_j - c_k \sum_i (f_i + f_k) c_i$$

where f_i and f_j represent the size-dependent components of the aggregation rate for clusters of magnitude i and j, respectively.

The normalized concentration of clusters of magnitude k, relative to the total number of clusters N, is denoted by ψ_k:

$$\psi_k = \frac{c_k}{N}$$

Differentiating with respect to the time variable t,

$$\dot{\psi_k} = \frac{\dot{c_k}}{N} - \frac{c_k \dot{N}}{N^2}$$

Substituting $\dot{N} = -N \sum_i f_i c_i$ into the equation $\dot{\psi_k}$,

$$\dot{\psi_k} = \frac{\dot{c_k}}{N} + \psi_k \sum_i f_i c_i$$

$$\dot{c_k} = N\dot{\psi_k} - N\psi_k \sum_i f_i c_i$$

Substituting $\dot{c_k}$ into the original equation:

$$N\dot{\psi_k} - N\psi_k \sum_i f_i c_i = \frac{1}{2} \sum_{i+j=k} (f_i + f_j) c_i c_j - c_k \sum_i (f_i + f_k) c_i$$

$$\dot{\psi}_k - \psi_k \sum_i f_i c_i = \frac{1}{2N} \sum_{i+j=k} (f_i + f_j) c_i c_j - \frac{c_k}{N} \sum_i (f_i + f_k) c_i$$

$$\dot{\psi}_k - \psi_k \sum_i f_i c_i = \frac{1}{2N} \sum_{i+j=k} (f_i + f_j)(\psi_i N)(\psi_j N) - \psi_k \sum_i (f_i + f_k)(\psi_i N)$$

$$\dot{\psi}_k - \psi_k \sum_i f_i(\psi_i N) = \frac{1}{2} \sum_{i+j=k} (f_i + f_j)\psi_i \psi_j N - \psi_k \sum_i (f_i + f_k)(\psi_i N)$$

$$\dot{\psi}_k = \frac{1}{2} \sum_{i+j=k} (f_i + f_j)\psi_i \psi_j N - \psi_k N f_k$$

$$\frac{\dot{\psi}_k}{N} = \frac{1}{2} \sum_{i+j=k} (f_i + f_j)\psi_i \psi_j - \psi_k f_k$$

Since the term $\frac{1}{2} \sum_{i+j=k} (f_i + f_j)\psi_i \psi_j$ is symmetric, it can be split into two identical sums,

$$\sum_{i+j=k} (f_i + f_j)\psi_i \psi_j = \sum_{i+j=k} f_i \psi_i \psi_j + \sum_{i+j=k} f_j \psi_i \psi_j$$

$$\sum_{i+j=k} f_i \psi_i \psi_j = \sum_{i+j=k} f_j \psi_i \psi_j$$

$$\sum_{i+j=k} (f_i + f_j)\psi_i \psi_j = 2 \sum_{i+j=k} f_i \psi_i \psi_j$$

$$\frac{\dot{\psi}_k}{N} = \sum_{i+j=k} f_i \psi_i \psi_j - \psi_k f_k$$

where the prime represents differentiation with respect to the rescaled time variable x, with $dt = dx/N$:

$$\psi'_k + f_k \psi_k = \sum_{i+j=k} f_i \psi_i \psi_j$$

The master equation is reformulated into the canonical Linear Differential Equation (LDE), with its integrating factor given by:

$$I.F. = \exp\left(\int f_k dx\right) = e^{kx}$$

Multiplying both sides of the LDE with the integrating factor:

$$e^{kx}\psi'_k + e^{kx} f_k \psi_k = e^{kx} \sum_{i+j=k} f_i \psi_i \psi_j$$

$$\frac{d}{dx}\left(e^{kx}\psi_k\right) = e^{kx}\sum_{i+j=k} i\psi_i\psi_j$$

Representing the $\Psi_k = I\psi_k = e^{kx}\psi_k$:

$$\Psi_k' = e^{kx}\sum_{i+j=k}\frac{\Psi_i}{e^{ix}}\frac{\Psi_j}{e^{jx}}$$

$$\Psi_k' = \sum_{i+j=k} i\Psi_i\Psi_j$$

Through the application of the almost-exponential ansatz $\Psi_k = a_k x^{k-1}$, the expression can be transformed into an algebraic recursion formula:

$$(k-1)a_k x^{k-2} = \sum_{i+j=k} ia_i x^{i-1} a_j x^{j-1} = \sum_{i+j=k} ia_i a_j x^{i+j-2} = \sum_{i+j=k} ia_i a_j x^{k-2}$$

$$(k-1)a_k = \sum_{i+j=k} ia_i a_j$$

Applying the generating function approach $A(z) = \sum_k a_k e^{kz}$:

$$\sum_k e^{kz}(k-1)a_k = \sum_k e^{kz}\sum_{i+j=k} ia_i a_j$$

By rearranging and using the Cauchy product for series:

$$\sum_k e^{kz}(k-1)a_k = \left(\sum_i ia_i e^{iz}\right)\left(\sum_j a_j e^{jz}\right)$$

$$\sum_k ke^{kz}a_k + \sum_k a_k e^{kz} = \left(\sum_i ia_i e^{iz}\right)\left(\sum_j a_j e^{jz}\right)$$

$$A'(z) + A(z) = A'(z)A(z)$$

$$A'(z) = \frac{A(z)}{1-A(z)}$$

Separating the variables and integrating both sides:

$$\int\left(\frac{1}{A(z)} - 1\right)dA(z) = \int dz$$

$$\int \frac{1}{A(z)} dA(z) - \int 1 dA(z) = \int dz$$

$$\ln A(z) - A(z) = z$$

$$\ln A(z) = z + A(z)$$

Exponentiating both sides:

$$e^{\ln A(z)} = e^{z+A(z)}$$

$$A(z) = e^{z} \cdot e^{A(z)}$$

$$A(z)e^{-A(z)} = e^{z}$$

With the application of the Lagrange inversion formula, $A(z)$ becomes:

$$A(z) = \sum_{k=1}^{\infty} \frac{k^{n-1}}{k!} e^{kz}$$

Comparing with the generating function $A(z) = \sum_k a_k e^{kz}$:

$$a_k = \frac{k^{k-1}}{k!}$$

By substituting a_k into the almost-exponential ansatz function and correlating it with the normalized concentration, followed by converting the rescaled time variable to the original time variable, the instantaneous concentration of cluster magnitude k is determined as follows:

$$c_k(t) = \frac{k^{k-1}}{k!} (1 - e^{-t})^{k-1} e^{-t} e^{-k(1-e^{-t})}$$

$$\sum_k c_k(t) = \sum_k \frac{k^{k-1}}{k!} (1 - e^{-t})^{k-1} e^{-t} e^{-k(1-e^{-t})}$$

The sum $\sum_k c_k(t)$ represents the total number of instantaneous clusters, $N(t)$, which corresponds to the first moment and is equal to e^{-t}.

Thus,

$$N(t) = \sum_k c_k(t) = \sum_k \frac{k^{k-1}}{k!} (1 - e^{-t})^{k-1} e^{-k(1-e^{-t})} e^{-t} = e^{-t}$$

$$N(t) = e^{-t}$$

Molecular Electrostatic Potential (MEP)

The spatial charge distribution of DMDODGA induces a molecular electrostatic potential (MEP), which influences intermolecular interactions and reactivity (see Figure S2, left). MEP mapping enables visualization surface charge variations, facilitating the identification of electronegative regions and potential interaction sites [5]. The MEP surface was calculated using DFT, with solvation effects modeled by the Integral Equation Formalism Polarizable Continuum Model (IEFPCM), and the solute cavity defined by overlapping spheres in a homogeneous dielectric field ($\epsilon = 2.006$) to simulate the implicit n-dodecane environment.

The color scale spans from dark blue, representing electron-deficient regions prone to nucleophilic attack, to deep red, denoting electron-rich regions with a high propensity for electrophilic attack. The dark red regions on the MEP surface correspond to the carbonyl groups in DMDODGA, indicating zones of minimal electrostatic potential and high electron density, which suggest the presence of loosely bound or excess electrons. Following the carbonyl groups, the ether oxygen atom displays notable electron density on the MEP surface. Geometry optimization indicates the carbonyl groups are oriented oppositely to maximize separation, reducing electrostatic repulsion to enhance molecular stability. This spatial configuration, coupled with reduced steric hindrance around the carbonyl groups in DMDODGA relative to DGA analogs, enhances accessibility to polar entities and is anticipated to promote its cluster formation.

Homogenous DMDODGA: OPLS-AA Assessment

The accuracy and reliability of physical property predictions in MD simulations require force field validation, typically achieved by comparing simulation results with experimental data to confirm applicability and improve predictive reliability. Although OPLS-AA has been applied to other organic complexants, validation for DMDODGA was necessary. To this end, a homogeneous configuration of 1000 molecules was simulated at 1 bar and 298.15 K, followed by a 2 ns equilibration and a 30 ns production run. Theoretical density calculations, sampled at 5 ps intervals during the final 20 ns, produced an average density of 0.9535 g/cm^3, corresponding well with the experimental value of 0.948 g/cm^3 and showing minimal sensitivity to system size variations (see Figure S2, right). While further modifications to the force field could yield better agreement with empirical measurements, such adjustments would require an expanded experimental dataset for verification. The observed consistency is thus considered sufficient to validate the OPLS-AA use in subsequent simulations.

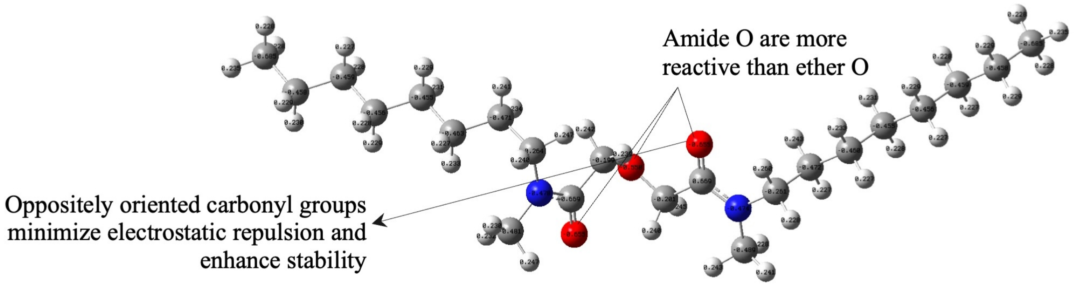

Figure S1: Charge Distribution - DMDODGA

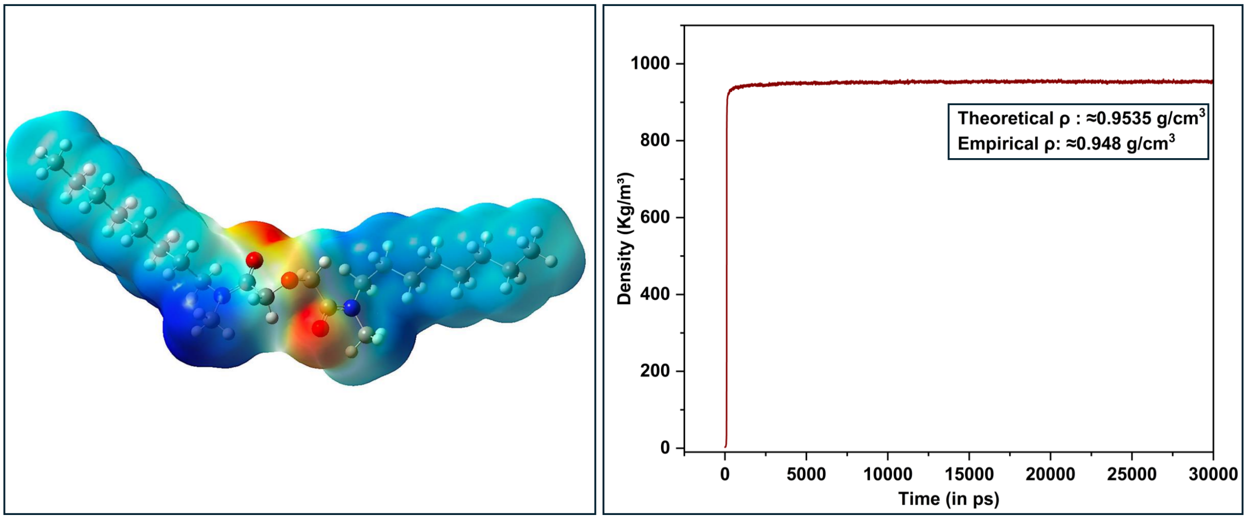

Figure S2: Left: Molecular electrostatic potential (MEP) surface of DMDODGA. The color gradient ranging from red to blue indicates the transition from zones of low to high electrostatic potential. Right: Density convergence profile of DMDODGA, simulated with OPLS-AA force field.

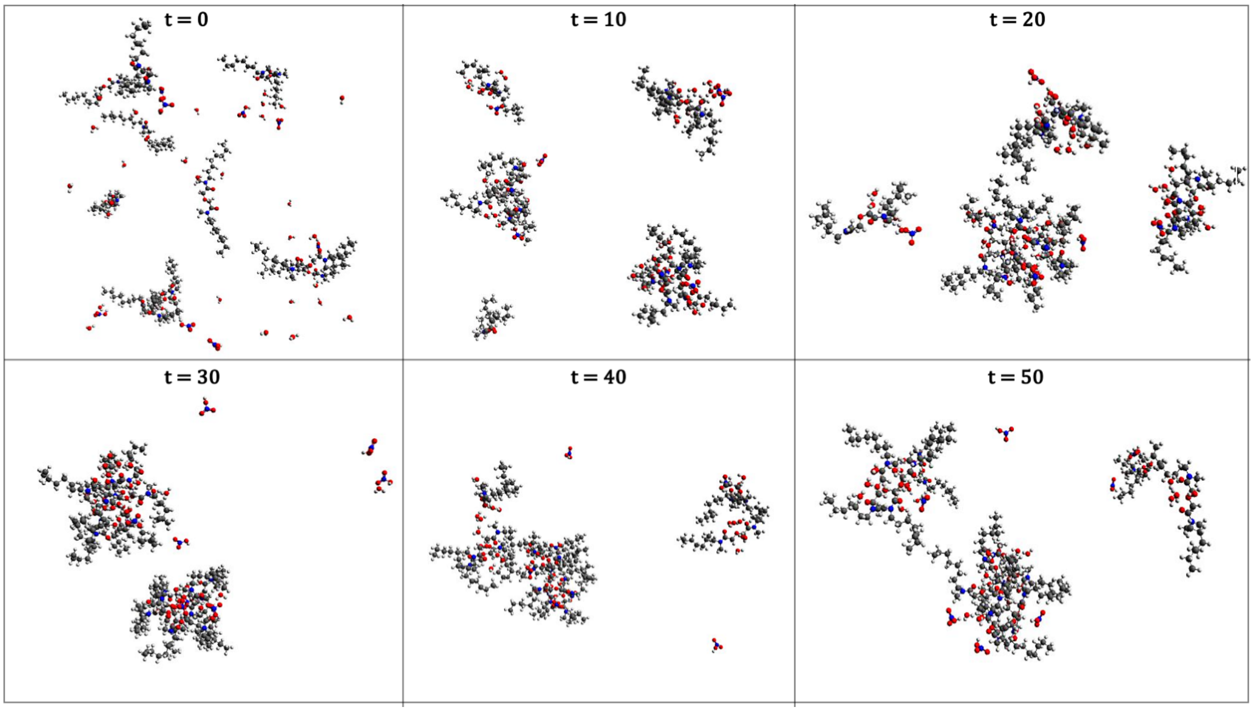

Figure S3: Evolution of Molecular Clusters in System 5: Sequential MD snapshots depicting configurational transitions over 0–50 ns.

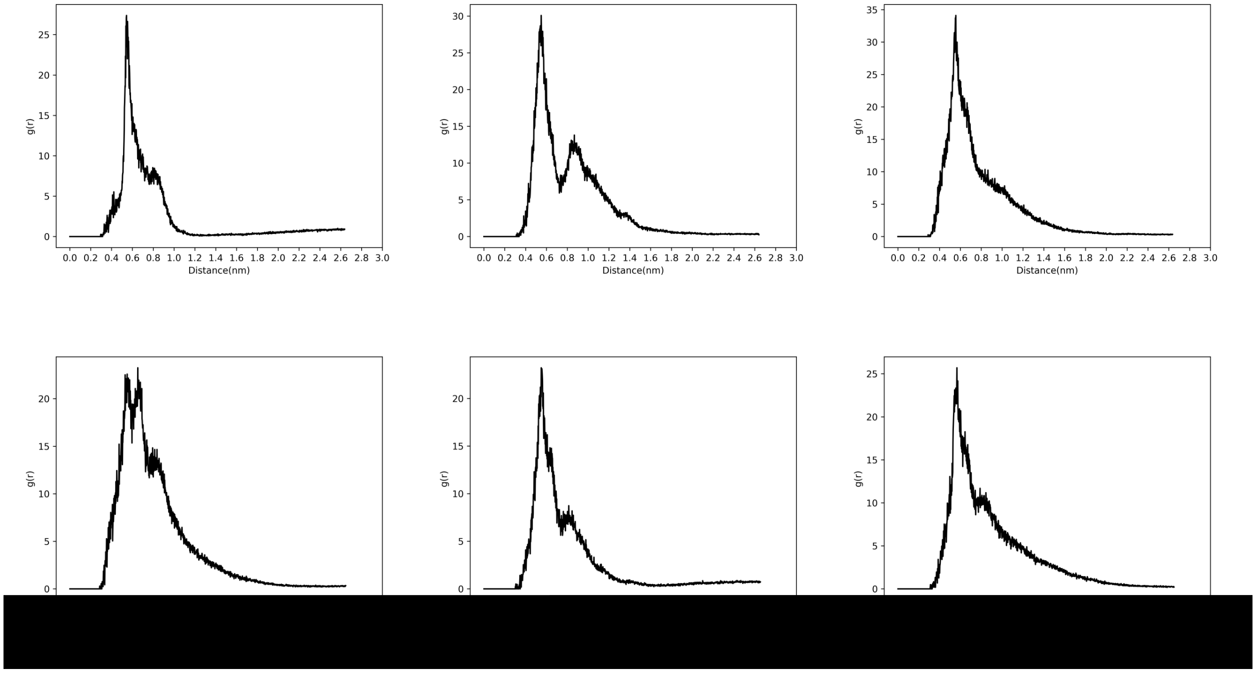

Figure S4: Radial distribution functions depicting DMDODGA-DMDODGA (carbonyl O) interactions. Top: Systems 1–3; Bottom: Systems 4–6.

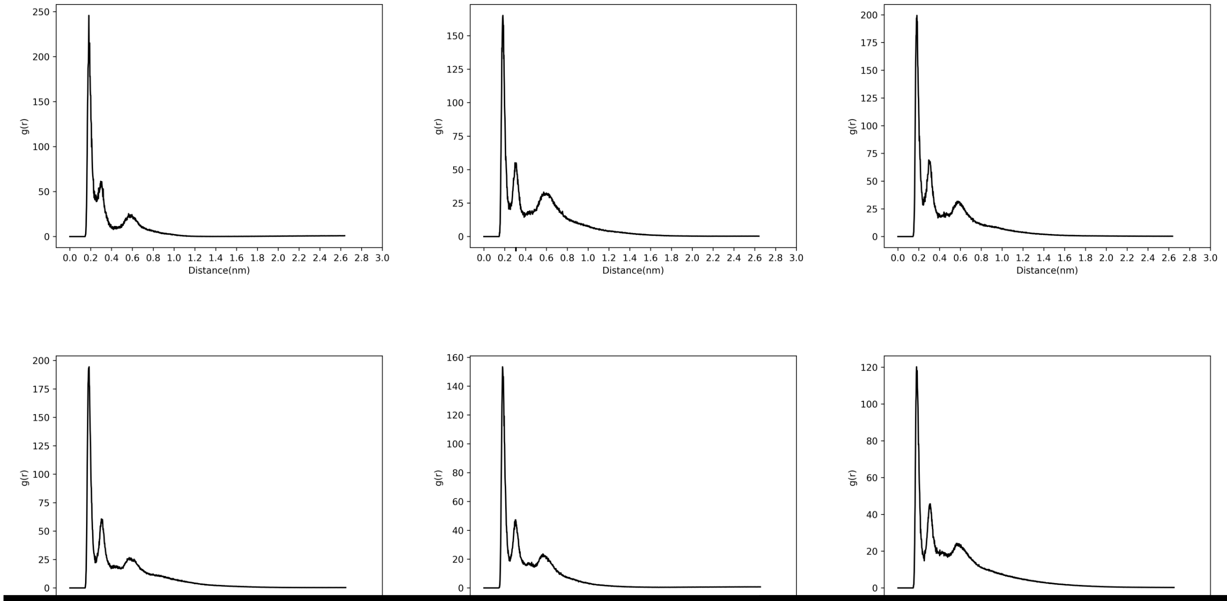

[redacted]

Figure S5: Radial distribution functions depicting DMDODGA-Water interactions. Top: Systems 1–3; Bottom: Systems 4–6.

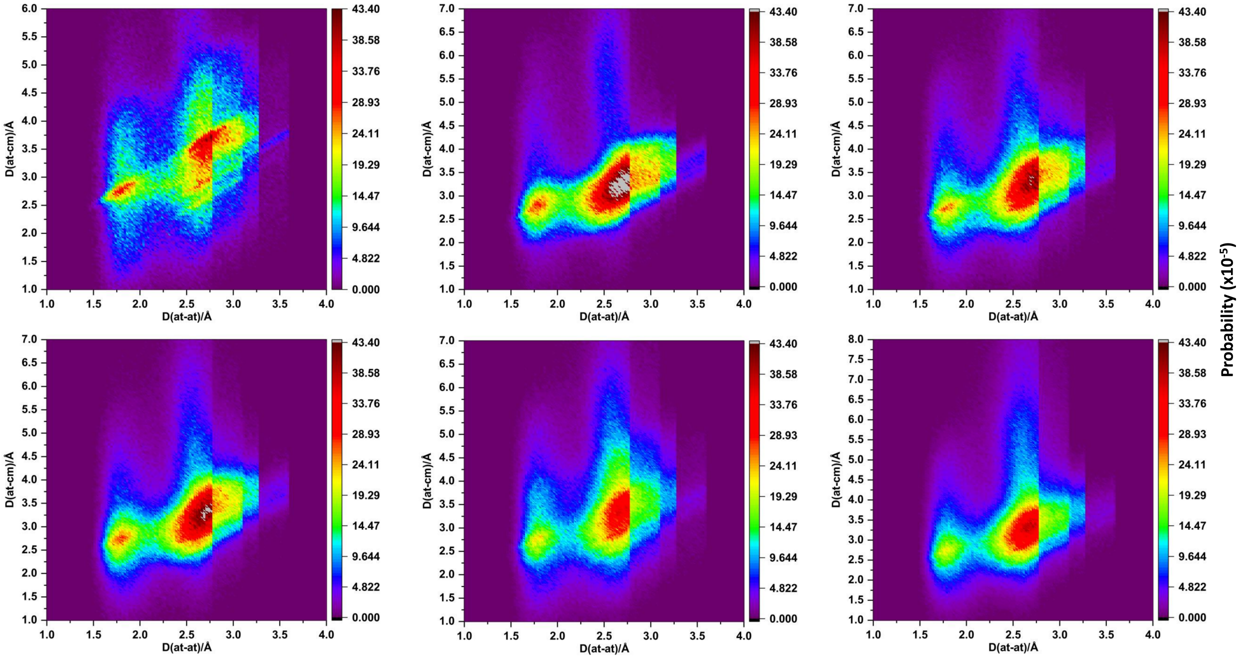

Figure S6: FSA of DMDODGA-Water. Top: Systems 1–3; Bottom: Systems 4–6.

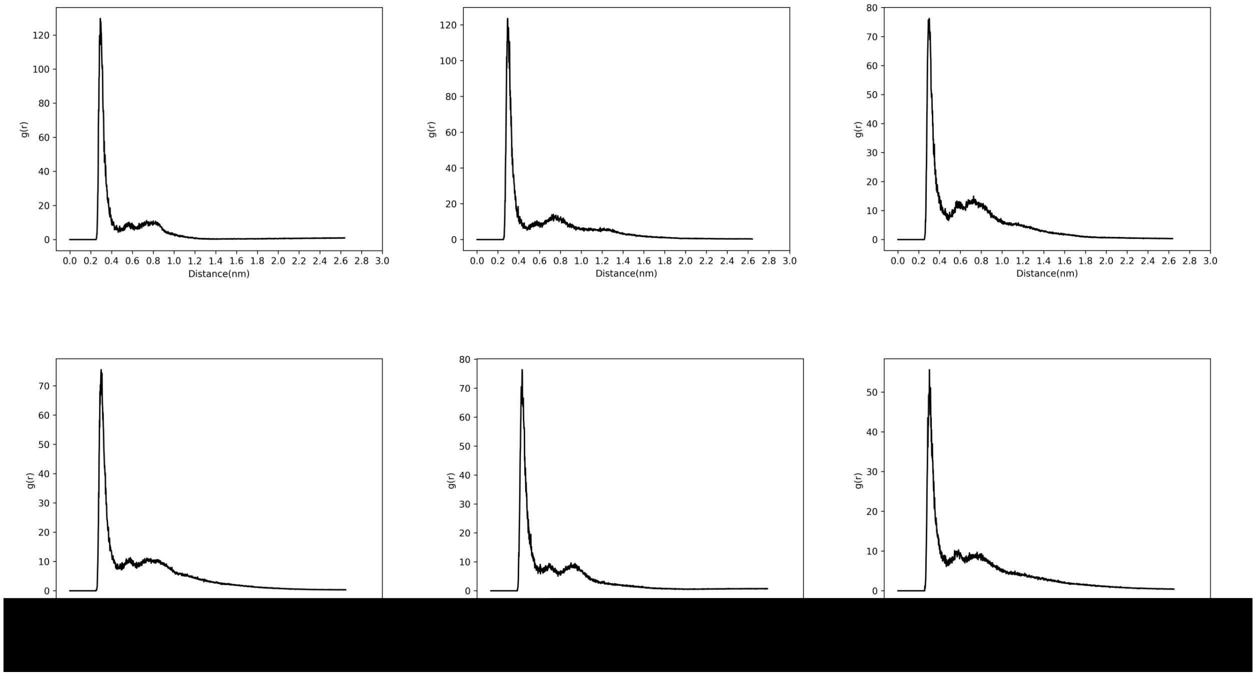

[redacted]

Figure S7: Radial distribution functions depicting DMDODGA-Nitric Acid interactions. Top: Systems 1–3; Bottom: Systems 4–6.

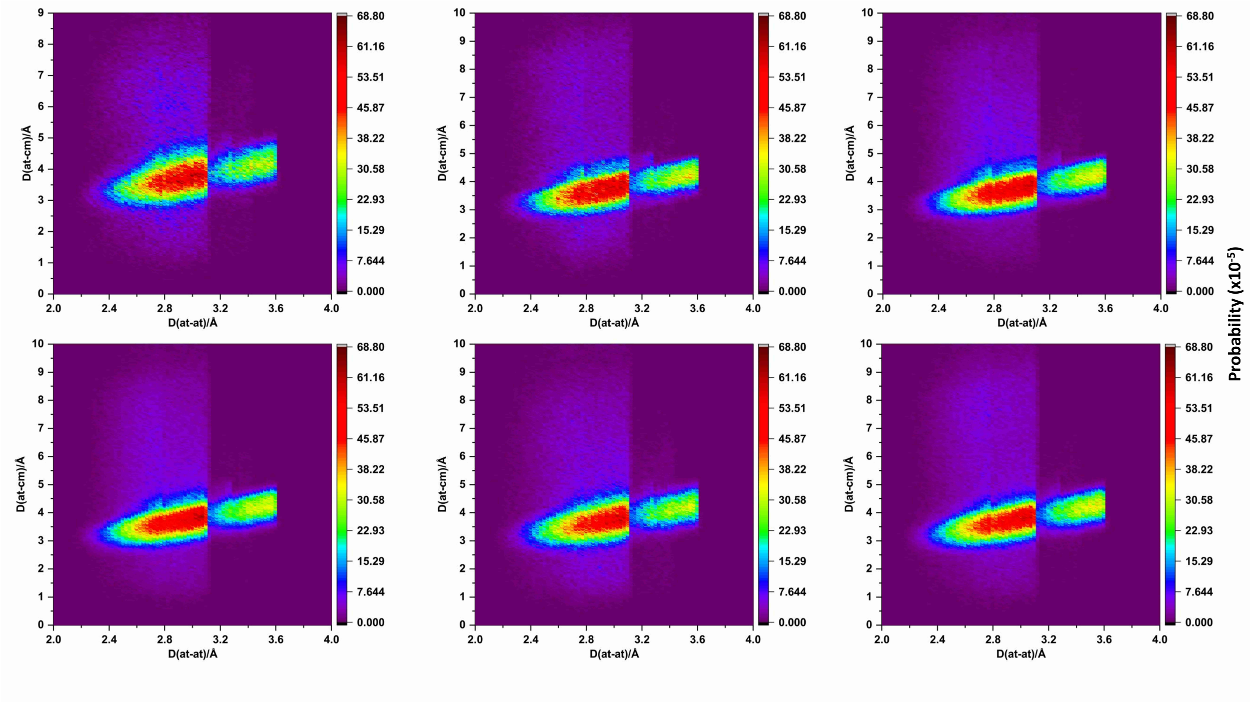

Figure S8: FSA of DMDODGA-Nitric Acid. Top: Systems 1–3; Bottom: Systems 4–6.

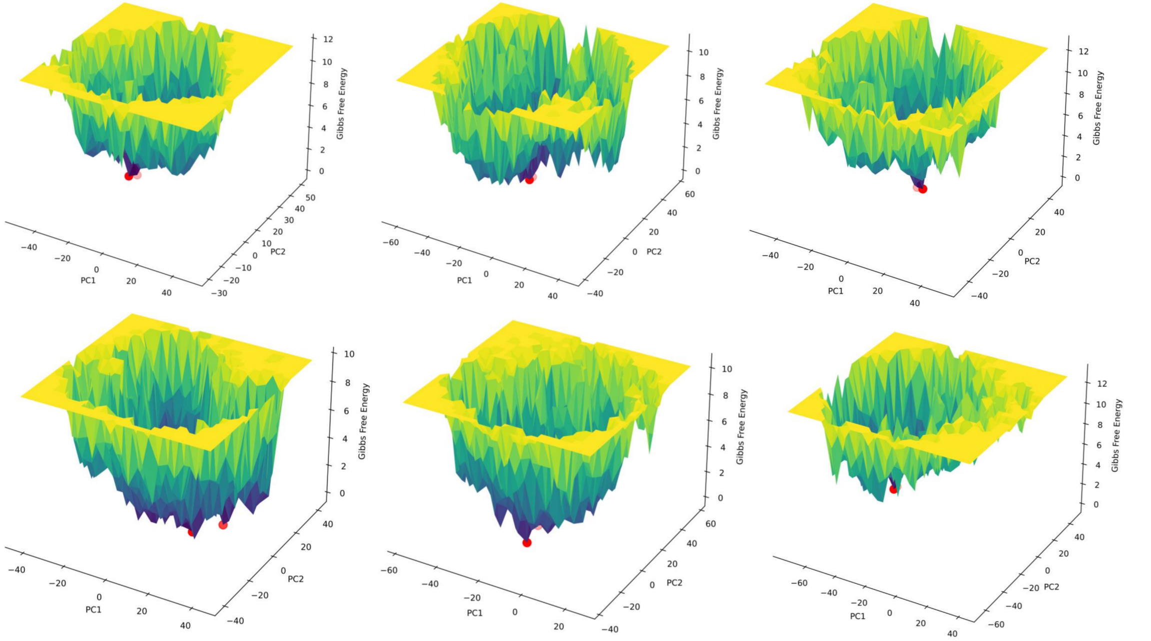

Figure S9: 3D free energy landscapes (FELs) depicting aggregation behavior. Top: Systems 1–3; Bottom: Systems 4–6.

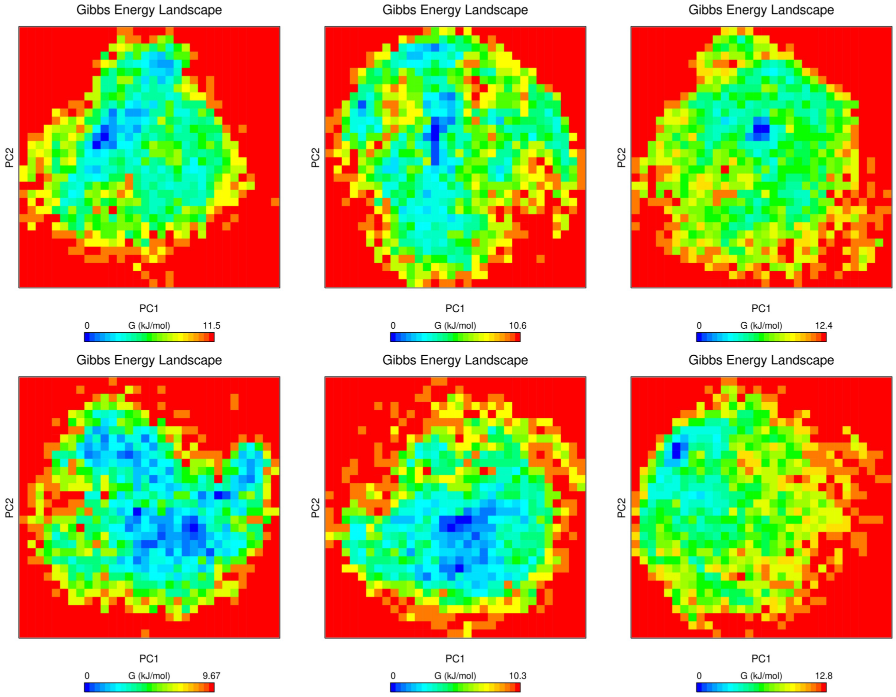

Figure S10: 2D mapping of free energy landscapes (FELs). Top: Systems 1–3; Bottom: Systems 4–6.

System Index	Equilibrium Constant (K)	$\Delta G_{\text{Isodesmic Model}}$ (kJ/mol)	Combined Probability Occurrence (Excluding Monomers/Dimers)	Weighted Average Cluster Size	ΔG_{PICFE} (kJ/mol)
1	12.03	-6.17	0.82	3.56	-4.90
2	7.93	-5.13	0.87	8.12	-2.64
3	16.00	-6.87	0.90	8.74	-4.55
4	9.70	-5.63	0.84	8.12	-2.65
5	10.37	-5.80	0.76	4.98	-2.57
6	19.29	-7.34	0.87	8.64	-4.67

Figure S11: Equilibrium constants, Gibbs free energy (ΔG) values from PICFE and Isodesmic models, and other cluster metrics across systems.

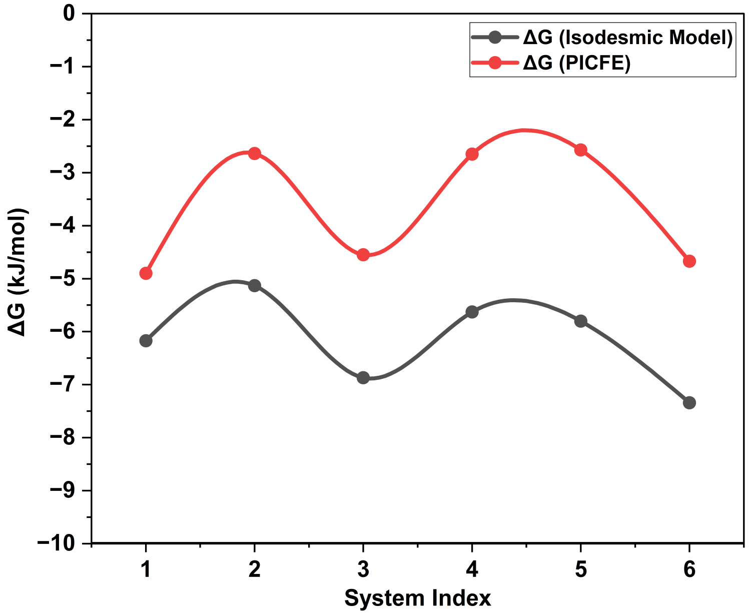

Figure S12: Comparative Aggregation Free Energy Analysis: ΔG values across all systems evaluated using Isodesmic and PICFE approaches.

Quantification of Cluster Stability via PICFE

In biphasic multicomponent extraction systems, the organic phase is often modeled under a dilute gas approximation. This simplification is widely used to characterize complexation structures and inform flowsheet modeling in solvent extraction. However, in industrial settings, the assumption breaks down due to high metal and extractant loadings, introducing significant non-ideality. This non-ideality is manifested through the formation of aggregates or cluster distributions within the organic phase. Incorporating such heterogeneous clustering into extraction models is challenging, given the metastable, labile, and disordered nature of these assemblies. Nevertheless, mesoscopic modeling frameworks provide a pathway to include cluster distributions for predictive simulations. In this context, thermodynamic free energy calculations are essential for estimating the chemical potentials associated with clusters during the mesoscopic stage of multiscale modeling.

To estimate the Gibbs free energy of clusters, the probability-based invariant cluster free energy (PICFE) approach can be employed to account for the effects of cluster size distribution within the system. This method emphasizes predominant aggregates, which largely govern the thermodynamic stability of the overall cluster population. Starting from a generalized modeling framework, PICFE adapts to system-specific requirements. In this formulation, the total partition function for a given cluster size is derived from a multinomial expansion of the grand canonical ensemble and is subsequently used to compute the molecular partition function for each cluster magnitude. The established correlation between the total and molecular partition functions facilitates the evaluation of the Helmholtz free energy, which subsequently defines the relationship between the chemical potential of clusters of size i through their molecular partition functions.

The grand partition function, often denoted by Ξ, is a generating function that sums over the entire compositional states:

$$\Xi(\lambda, V, T) = \sum_{N_i=0}^{\infty} \lambda^{N_i} Q(N_i, V, T) \tag{7}$$

where $\lambda = e^{\beta\mu}$ is the activity, with $\beta = \frac{1}{k_B T}$ and μ being the chemical potential. $Q(N_i, V, T)$ represents the total partition function.

To avoid generating all partitions of N_i, at fixed volume V and temperature T, $Q(N_i)$ is derived as the coefficient of λ^{N_i} in the expansion of the grand partition function $\Xi(\lambda)$ [6]:

$$\Xi(\lambda) = \sum_{N_i=0}^{\infty} \lambda^{N_i} Q(N_i) = \exp\left(\sum_{i=1}^{\infty} q_i \lambda^i\right) = \sum_{n=0}^{\infty} \frac{1}{n!} \left(\sum_{i=1}^{\infty} q_i \lambda^i\right)^n \tag{8}$$

where q_i represents the single-particle partition function for state i.

By computing the N_i-th order derivative of the grand partition function's series expansion at $\lambda = 0$, the coefficient of λ^{N_i} is obtained as follows:

$$Q(N_i) = \frac{1}{N_i!}\frac{d^{N_i}}{d\lambda^{N_i}} \exp\left(\sum_{i=1}^{\infty} q_i \lambda^i\right)\Bigg|_{\lambda=0} = \sum_{\substack{k_1+k_2+\ldots+k_m=n \\ k_1+2k_2+\cdots+mk_m=N_i}} \frac{q_1^{k_1} q_2^{k_2}\cdots q_m^{k_m}}{k_1!k_2!\cdots k_m!} \tag{9}$$

For a particular state i, under conditions of constant V and T, the coefficient simplifies to the following expression:

$$Q = Q(N_i) = \frac{(q_i)^{N_i}}{N_i!} \tag{10}$$

The Helmholtz free energy can be expressed as a function of the total partition function as follows:

$$F = -k_B T \ln Q = -k_B T \ln\left(\frac{(q_i)^{N_i}}{N_i!}\right) \tag{11}$$

where F is the Helmholtz free energy, and N_i represents the number of particles in state i.

Subsequently, the chemical potential can be determined from the Helmholtz free energy using the following relationship:

$$\mu_i = \left(\frac{\partial F}{\partial N_i}\right)_{T,V} = \left(\frac{\partial(-k_B T \ln\left(\frac{(q_i)^{N_i}}{N_i!}\right))}{\partial N_i}\right)_{T,V} \tag{12}$$

where μ_i is the chemical potential of state i.

Applying Stirling's approximation, $\ln(N_i!) \approx N_i \ln N_i - N_i$, and subsequently solving the resulting expression, the relationship between the Helmholtz free energy and the molecular partition function is obtained as follows:

$$\mu_i = \left(\frac{\partial F}{\partial N_i}\right)_{T,V} = -k_B T \ln\left(\frac{q_i}{N_i}\right) \tag{13}$$

Within the theoretical framework of Henry's Law, the chemical potential of species i, μ_i, can be correlated to the concentration of the corresponding cluster as follows:

$$\mu_i = \mu_i^\circ + k_B T \ln\left(\frac{a}{a^\circ}\right) = -k_B T \ln\left(\frac{q_i}{N_i}\right) + k_B T \ln\left(\frac{c}{c^\circ}\right) \tag{14}$$

where μ_i° is the standard chemical potential, a and a° denote the activity and standard activity, respectively, and c and c° represent the concentration and standard concentration.

The Gibbs free energy change associated with the formation of an i-magnitude cluster from i individual monomers, under constant T and pressure P, is represented by:

$$\Delta G_i = \mu_i - i\mu_1 \tag{15}$$

where ΔG_i is the Gibbs free energy change, μ_i is the chemical potential of the i-magnitude cluster, and μ_1 is the chemical potential of a monomeric state.

Assuming the concentrations of both monomers and i-sized clusters are described by c [6], and substituting the expression for μ_i into the equation for ΔG_i:

$$-k_B T \ln\left(\frac{q_i}{q_1^i}\right) = \mu_i^o - i\mu_1^o + k_B T(1-i)\ln\left(\frac{c}{c^\circ}\right)$$

$$\Delta G_i = \Delta G_i^o - k_B T(i-1)\ln\left(\frac{c}{c^\circ}\right)$$

The standard state Gibbs free energy change, ΔG_i^o, for an i-sized cluster can be related to the local equilibrium association constant K_i as:

$$\Delta G_i^\circ = -k_B T \ln K_i \tag{16}$$

Consequently, the equation can be expressed as follows:

$$\Delta G_i = -k_B T\left[\ln K_i + \ln\left(\frac{c}{c^\circ}\right)^{i-1}\right] \tag{17}$$

Due to its dependence on local association constants and concentration, the equation is primarily applicable to Gibbs free energy calculations of single-sized aggregates. While it can be used directly if the full aggregate distribution and composition are known, this restricts broader energetic evaluation—particularly in systems where a specific range of aggregate sizes dominates. In such cases, where certain aggregates define the overall distribution, the law of mass action can be applied to extend the cluster-specific framework to a distribution-wide model for computing global free energy. This extension involves two key approximations. First, the system association constant is derived under the assumption that the Gibbs free energy change associated with adding an additional residue remains invariant with cluster size i, consistent with the isodesmic model approximation [7]. Second, the equilibrium probability occurrences of DMDODGA clusters are used to estimate individual cluster concentrations and are well within the scope of molecular dynamics simulations.

The derivation of the system association constant, illustrating the first approximation, is presented below:

$$M_{n-1} + M_1 \leftrightarrow M_n$$

$$K = \frac{\{M_n\}}{\{M_{n-1}\}\{M_1\}} = \frac{\lambda_n}{\lambda_{n-1}\lambda_1}$$

$$\lambda_n = K\lambda_{n-1}\lambda_1$$

$$\lambda_n = K^{n-1}\lambda_1^n$$

$$K = e^{\frac{\ln(\lambda_n) - n\ln(\lambda_1)}{n-1}} \tag{18}$$

where K denotes the representative system association constant and λ represents the activity coefficient. The analysis specifically excludes monomers and dimers due to their negligible contribution to the stabilization of larger clusters. With the parameters (K, c) now representing system-wide metrics, the PICFE approach defines the cluster size i as representative of the overall system through the introduction of the weighted average aggregate size. By incorporating the system association constant, weighted average cluster size, and concentrations based on the second approximation into equation 17, the resulting formulation becomes:

$$\frac{\Delta G_i}{k_B T} = -\ln\left(\frac{\lambda_n}{\lambda_1}\right)^{\frac{1}{n-1}} - \left(\frac{\sum_{i=3}^{N}(i \times P_i)}{\sum_{i=3}^{N} P_i} - 1\right)\ln\left(\frac{\sum_{i=3}^{N}(P_i \times c^0)}{c^0}\right) \tag{19}$$

where P_i denotes the probability of clusters with magnitude i. Probability occurrences for the respective cluster magnitudes are used relative to the reference concentration c^0. This equation can be directly applied to evaluate the system-wide thermodynamics, provided the representative association constant and probabilistic cluster distribution are known. The resulting free energy of the aggregate distribution can then be incorporated into mesoscopic modeling of the pseudophases in extraction processes.

References

[1] Carlos E. S. Bernardes. AGGREGATES: Finding structures in simulation results of solutions. *Journal of Computational Chemistry*, 38(10):753–765, 2017.

[2] Andrea Amadei, Antonius B. M. Linssen, and Herman J. C. Berendsen. Essential dynamics of proteins. *Proteins: Structure, Function, and Bioinformatics*, 17(4):412–425, 1993.

[3] Charles C. David and Donald J. Jacobs. Principal Component Analysis: A Method for Determining the Essential Dynamics of Proteins. *Methods in molecular biology (Clifton, N.J.)*, 1084:193–226, 2014.

[4] Pavel L. Krapivsky, Sidney Redner, and Eli Ben-Naim. *Aggregation*, page 134–171. Cambridge University Press, 2010.

[5] Peter Politzer and Donald G. Truhlar. *Chemical Applications of Atomic and Molecular Electrostatic Potentials: Reactivity, Structure, Scattering, and Energetics of Organic, Inorganic, and Biological Systems*. Springer Science & Business Media, June 2013.

[6] Xiaokun Zhang, Jorge G. Arce Nunez, and James T. Kindt. Derivation of micelle size-dependent free energies of aggregation for octyl phosphocholine from molecular dynamics simulation. *Fluid Phase Equilibria*, 485:83–93, April 2019.

[7] Daniel Massey, Andrew Masters, Jonathan Macdonald-Taylor, David Woodhead, and Robin Taylor. Molecular Dynamics Study of the Aggregation Behavior of N,N,N′,N′-Tetraoctyl Diglycolamide. *J. Phys. Chem. B*, 126(33):6290–6300, August 2022.
